# Visual analysis of biological data-knowledge networks

**DOI:** 10.1186/s12859-015-0550-z

**Published:** 2015-04-29

**Authors:** Corinna Vehlow, David P Kao, Michael R Bristow, Lawrence E Hunter, Daniel Weiskopf, Carsten Görg

**Affiliations:** 1 VISUS, University of Stuttgart, Allmandring 19, Stuttgart, Germany; 2 School of Medicine, University of Colorado, E 17th Pl, Aurora, CO USA

**Keywords:** Network analysis, Degree-of-interest functions, Interactive visualization

## Abstract

**Background:**

The interpretation of the results from genome-scale experiments is a challenging and important problem in contemporary biomedical research. Biological networks that integrate experimental results with existing knowledge from biomedical databases and published literature can provide a rich resource and powerful basis for hypothesizing about mechanistic explanations for observed gene-phenotype relationships. However, the size and density of such networks often impede their efficient exploration and understanding.

**Results:**

We introduce a visual analytics approach that integrates interactive filtering of dense networks based on degree-of-interest functions with attribute-based layouts of the resulting subnetworks. The comparison of multiple subnetworks representing different analysis facets is facilitated through an interactive super-network that integrates brushing-and-linking techniques for highlighting components across networks. An implementation is freely available as a Cytoscape app.

**Conclusions:**

We demonstrate the utility of our approach through two case studies using a dataset that combines clinical data with high-throughput data for studying the effect of *β*-blocker treatment on heart failure patients. Furthermore, we discuss our team-based iterative design and development process as well as the limitations and generalizability of our approach.

**Electronic supplementary material:**

The online version of this article (doi:10.1186/s12859-015-0550-z) contains supplementary material, which is available to authorized users.

## Background

The interpretation of the results from genome-scale experiments is a challenging and important problem in contemporary biomedical research. The main purpose of interrogating experimental systems at genomic scale is to identify previously unsuspected entities and mechanisms underlying important biological phenomena. Often, such experiments identify hundreds or even thousands of previously unsuspected polymorphisms, gene products, metabolites, or other entities that play a role in phenotypes of interest. Hypothesizing about how and why the entities identified by experiment are related to the phenotype is a critical step in taking advantage of genome-scale technology to gain insights into life and to improve human health. Understanding and contextualizing results involving hundreds of entities can involve finding, integrating, and assimilating information scattered across potentially dozens of databases and thousands of publications, arising from many different disciplines and scientific communities.

One approach for analyzing these large and complex datasets is to create integrated data-knowledge networks that allow biomedical experts to analyze the results of an experiment in the context of existing knowledge from the literature and from databases. A recent article in the Science Policy Forum on amplifying scientific discovery with artificial intelligence [1] argues that the current human bottleneck in scientific discovery could be reduced by “systems [that] use encoded knowledge of scientific domains and processes in order to assist analysts with tasks that previously required human knowledge and reasoning”. The Hanalyzer (high-throughput analyzer) [2] was one of the first systems to support such a knowledge-based genome-scale interpretation approach. It was designed around biologist end-user needs as a mechanism for deploying knowledge-based computational tools to facilitate the analysis of genome-scale datasets. Hanalyzer has been used to interpret gene expression mircorarray results in diverse research areas, including craniofacial development [2], a mouse model type I diabetes [3], and human heart failure [4].

Integrated data-knowledge networks provide a rich resource and powerful basis for analyzing genome-scale data; however, the networks are difficult to explore and understand since they are overwhelmingly complex. Even when focusing on pre-specified hypotheses of the underlying experiment, the resulting networks often contain tens of thousands of interactions and relationships. Using current systems and interfaces, discussed at the end of this section, it can take months and considerable specialized expertise to “untangle the hairball” and identify subnetworks that represent plausible mechanistic explanations for observed biological phenomena. Through a longitudinal study [5] and collaborations with current users of the Hanalyzer prototype we identified two major limitations in the existing analysis workflow: (1) the integrated data-knowledge networks in Hanalyzer are rigid and difficult to navigate, and (2) the manual analysis process of the very dense integrated data-knowledge networks is very slow and cumbersome.

As our first contribution, we present a set of innovative approaches using degree-of-interest (DoI) functions to overcome these limitations; they include DoI-based filtering, graph layout, and a network comparison technique. In combination, they support the analysis of integrated data-knowledge networks and help domain experts to discover new insights and to establish novel hypotheses. The DoI functions are sensitive to the current state of the knowledge, the particulars of the experiment that generated the data being analyzed, and to the specific analyst who explores the data. Our second contribution is the design and development of the *RenoDoI* framework, an application to untangle (lt. renodare) large and dense networks using DoI functions, and its integration in the network visualization framework Cytoscape [6]. We demonstrate the utility of the *RenoDoI* framework through two case studies using a dataset from a clinical trial investigating different drug treatments for heart failure patients.

### Biomedical data and analysis tasks

Our approach supports the analysis of rich biomedical datasets that can include clinical data, transcriptomic profiling data, and knowledge-based data. Clinical data refers to tests performed on patients to establish phenotypes and can also include multiple drug treatment groups. Transcriptomic profiling data refers to the measurement of gene expression, for example through quantifying mRNA fragments that encode proteins. Knowledge-based data refers to existing databases and ontologies with curated biomedical knowledge as well as the literature. We will discuss one instance of biomedical data in detail in our case studies in the Results Section.

We developed *RenoDoI* through an iterative design process. Our team consists of visualization experts and biomedical researchers from the Division of Cardiology in the CU Medical School. Through this interdisciplinary work, we were able to develop a deep understanding of analysis needs and include regular feedback on the successive prototypes of our tool. Analyzing signaling pathways and biological function are critical components of our primary analysis goals. During our iterative design process, we expanded these goals to more detailed and specific tasks to answer the following key biological questions.

**I: Find genes and gene-gene correlations associated with phenotype of interest** Given the high-dimensional nature of transcriptomic profiling data, it is often useful to be able to view candidate genes and gene-gene associations according to analyst-specified significance thresholds. This task allows for the selection of experimental data subsets dynamically based on strength association with specific phenotypes.

**II: Identify biological relationships among genes associated with the phenotype of interest** The first step in developing a general hypothesis to explain patterns of gene expression associated with the phenotype of interest is determining whether genes associated with the phenotype have shared biological properties. This is accomplished by combining knowledge from curated databases and the biomedical literature with relationships identified in the experimental data and identifying biological processes that are represented by statistically significant genes.

**III: Identify genes that overlap between multiple signaling pathways or biological processes that may be critical for a specific phenotype** The large number of “interesting” candidate genes makes conventional analysis to identify “hub” genes that affect multiple biological processes in a table-like format challenging. Identification of critical genes that regulate molecular and phenotypic changes requires understanding of relationships between genes that share biological characteristics.

**IV: Identify specific gene-gene correlations in experimental data supported by existing knowledge derived from analyst-specified knowledge sources** Elucidating the mechanism of experimental findings can be challenging when the nature of the relationships between “interesting” genes is not yet known. In such situations, viewing combined knowledge and experimental data using n knowledge sources (e.g., signaling pathways or biological functions) can facilitate the development of hypotheses regarding the primary relationship between interesting genes.

**V: Compare integrated data-knowledge networks derived using different knowledge sources** Single knowledge sources may be incomplete or limited in scope (e.g., functional pathways vs. cellular compartments). Direct comparison of analyst-specified single knowledge sources similar in scope may help determine the degree of support for an annotation. Comparison of overlap between knowledge between sources with complementary scope may enhance biological understanding of gene-gene relationships and may help identify novel hypotheses (e.g., functional pathways and drug target knowledge sources).

To support an analyst working on these identified tasks we integrated three analytical elements in one workflow. As the aforementioned integrated data-knowledge networks are not only large but also very dense, it is difficult to generate meaningful layouts for these node-link diagrams. Therefore, *RenoDoI* first allows analysts to quickly filter a complex network to a small set of relevant nodes and edges by combining multiple DoI functions. As a second analytical step, we developed an interactive layout approach that (semi-)automatically groups and displays the nodes of the extracted task-specific subnetworks according to a user-defined subset of node attributes. The analyst can influence the resulting layout so that it reflects how she thinks about the specific subnetwork in the context of the given task. As a third analysis step we support the comparison of multiple extracted subnetworks to help analysts examining the data in the context of different knowledge sources or phenotypes of interest.

In the remainder of this section, we first discuss existing tools and approaches for knowledge-based analyses and then review the related work on the application of DoI functions to networks, set system visualization, and network comparison techniques.

**Knowledge-based visual analytics** There exist several approaches that can facilitate the knowledge-based analysis of results from genome-scale experiments, however, only few are based on a human-centered visual analysis approach. Traditionally, researchers use statistical approaches to derive a relatively small set of genes from experimental results, and then attempt to study these genes through manual collection and integration of knowledge from various curated databases and the literature. This approach is slow and labor intensive and, therefore, only a small set of genes can be analyzed.

Another set of approaches uses a variety of enrichment techniques. They attempt to characterize the functional classes that are overrepresented in a large group of genes. Huang et al. [7] reviewed 68 enrichment tools and classified them into three categories: (1) singular enrichment analysis tools that start with a preselected list of interesting genes and test the enrichment of each annotation term in a linear mode, (2) gene set enrichment analysis tools that take all genes from a genome-scale experiment into account without selecting significant genes, and (3) modular enrichment analysis tools that include additional network discovery algorithms by considering term-to-term relationships. Prominent enrichment tools include the web-based applications DAVID [8] and GOEAST [9], and the Cytoscape PlugIn BiNGO [10]. Their common limitations include that functional annotations are often incomplete or over-focused, among others as discussed in [7].

Proprietary systems, such as the Ingenuity Pathway Assistant, are quite expensive and users often do not trust them since they act like a black box; it is not transparent what knowledge sources they contain and how the embedded algorithms work. The eXamine [11] app for Cytoscape [6] provides an approach for exploring small modules in biological networks that are annotated with various knowledge attributes. It uses a visual set-based approach (discussed in the set system visualization section below) to guide the analysis process and relies on a human analyst to explore the data and interpret the results (in contrast to the previous statistical-based approaches); it is the system most similar to our approach.

The Hanalyzer system [2] uses integrated data-knowledge networks as an analysis basis. It starts from a list of genes that were implicated in an experiment. Prior knowledge about those genes and their relationships to each other is extracted from 26 different knowledge sources (including pathways from KEGG [12] and Reactome [13], gene annotations from ontologies such as GO [14], and disease annotations from GAD [15]), plus co-occurrence in PubMed abstracts and certain types of inferred relationships. The knowledge extraction results are summarized in knowledge networks that are then superimposed on weighted data networks—usually gene-gene correlation networks. The Hanalyzer PlugIn for Cytoscape supports the user investigating associated annotations using a nested context menu. Browsing through all annotations for all elements is a tiresome task when searching for relevant genes and interactions. Our new tool *RenoDoI* works on the same network model as Hanalyzer but with advanced interaction and visualization capabilities.

**Application of DoI functions to networks** The concept of DoI functions is widely used in scientific visualization for focus-and-context techniques [16], such as space-distortion techniques, or highlighting based on blurring, opacity, or color. We differentiate between discrete and continuous (fuzzy) DoI functions, where the latter describe a gradual transition from the subset of interest to the rest. In graph drawing, numerous approaches exist for computing the DoI based on a user-defined focus. The user typically defines one or more nodes of interest, based on which the DoI is diffused over a tree [17] or graph [18,19]: less interesting parts of the tree or graph can be aggregated [17,18,20] or faded out using logical filtering [19,20]. In contrast to previous work, we do not derive the DoI based on the user’s selection since this approach does not facilitate our type of analysis. For our tasks, the analyst explores the data with the goal of identifying genes associated with a phenotype of interest and hence these genes are not known beforehand. Furthermore, we use the DoI functions not only to fade out uninteresting components, but also to rearrange the interesting components based on selected annotations. To our knowledge, this type of DoI approach has not yet been applied to the biological domain, and therefore, there exist no systems that are directly comparable to ours.

**Set system visualization.** One way to visualize set associations is to encoded the set memberships in the node positions. There exist several layout approaches that use set membership to guide the node placement in the plane but only few consider overlaps between sets. The approach by Zhang et al. [21] allows for the overlap of gene ontology annotations by duplicating nodes annotated to different sets and hence regions. This approach does not facilitate the task we want to address—identifying genes that overlap between multiple annotations (Task III). eXamine [11] models the set-based annotations, e.g., pathway and ontology terms, and relationships of a graph as a hyper-graph and uses a self-organized map approach to lay out the node-link diagram and contours surrounding the sets. The hybrid approach by Itoh et al. [22] represents clusters using a meta-graph that is laid out using a force-directed algorithm, where nodes within the clusters are positioned on a grid. In comparison to these approaches, our layout approach is semi-automatic and leaves the user the opportunity to intervene and adjust the arrangement of groups and their intersections to match the user’s mental map. Our approach is also different in regard to the stability of layouts. eXamine [11] adjusts the layout every time the selection and contour-based highlighting of annotations changes. The resulting new layout is derived from the previous layout to avoid severe changes in the new layout. This approach guarantees an optimal layout with respect to a few annotations but it is not possible to re-create the very same layout for the same annotations as the layout always depends on the initial position.

Independent from the layout, overlapping sets of nodes or elements are commonly represented using overlays including Euler-like diagrams or bounding isocontours in general, or region-, line-, or glyph-based overlays [23]. As we do not have a linear order of elements within each group and node color is used to convey experimental data information, line-based and glyph-based approaches are not suitable for our application. Instead, techniques such as Euler-like diagrams [24,25], overlapping convex hulls [26], or bounding isocontours [27] are more suitable.

**Network comparison techniques.** There are four general approaches for visual comparison that can also be applied to networks [28,29]: juxtaposition (showing objects side-by-side), superposition (overlaying objects), combinations of juxtaposition with superposition [30-32], and the explicit encoding of similarities and differences. Approaches that use juxtaposition or superposition to compare networks position nodes at similar relative positions [33] or even at the same positions derived from the super-graph [34]. However, the node positions themselves do not directly reveal which parts of the combined network belong to which of the initial networks that should be compared. Therefore, superpositions and juxtapositions are usually combined with the explicit encoding of the similarities and/or differences in the networks. Color, other visual attributes, or coordinated multiple views brushing [35] can be used as an encoding or interaction technique. However, the aforementioned approaches align biological networks [28,31] or single pathways [30,32,33,35] without considering the overlap of different annotations (e.g., pathways) available for the networks under investigation (Task III).

## Methods

To support analysts in working on the analysis tasks described in the previous section, we have developed *RenoDoI* and implemented it as an application in Cytoscape [6]. We now describe the underlying visualization methods and the implementation of the tool. *RenoDoI* integrates four visual analytics aspects:

- the DoI-based filtering of a large integrated data-knowledge network,
- the extraction and rearrangement of subnetworks with respect to knowledge-based annotation subsets,
- the comparison of two or more subnetworks,
- and the investigation of the underlying experimental data in the context of subnetworks.

**Figure 1:**
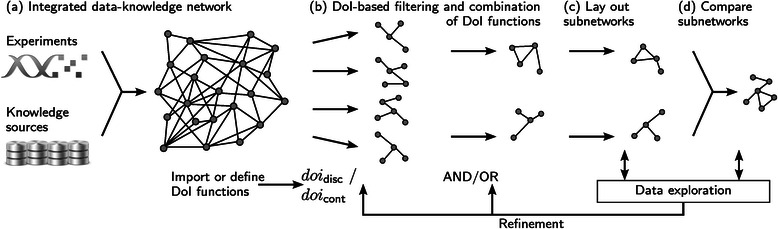
Schematic overview of our approach.**(a)** Combination of experimental data with knowledge to generate an integrated data-knowledge network. **(b)** Filter subnetworks based on continuous and/or discrete degree-of-interest (DoI) functions. **(c)** Lay out the subnetworks semi-automatically based on a user-selected set of interesting annotations. **(d)** Generate the super network for comparison.

A typical workflow involving these aspects is illustrated in Figure 1.

### Data model

We designed our approach for integrated data-knowledge networks. We model such a network *G*=(*V*,*E*) as a set of vertices *V* (here genes) and edges *E* (here different types of relationships between genes). Each gene (*v*_*i*_∈*V*) is associated with some transcriptomic profiling data, e.g., gene expression levels of different samples from multiple time points, treatment conditions, and subjects. Further knowledge about each of the genes *v*_*i*_ is derived from *k* ^′^ integrated knowledge sources $\mathcal {K}_{k}$. As discussed in the previous section on the Hanalyzer approach [2], the integrated data-knowledge network *G* is constructed by combining the data network with the knowledge network. An edge *e*_*j*_ may therefore represent data relationships (e.g, a statistically significant correlation between expression levels of the two connected genes), knowledge relationships (e.g., relationships derived from one or more of the integrated knowledge sources $\mathcal {K}_{k}$), or both.

Our approach uses two types of DoI functions: *continuous* and *discrete* DoI functions. In our application scenarios, the continuous DoI functions $\phantom {\dot {i}\!}\text {doi}_{\text {cont}_{l}}\in [0,1]$ are based on either node attributes or edge attributes interpreted as the DoI of vertices or edges, respectively, where *l* ^′^ is the number of available continuous DoI functions. Our DoI functions have the following semantic meaning: higher values are interpreted as more interesting, i.e., the higher $\phantom {\dot {i}\!}\text {doi}_{\text {cont}_{l}}$, the more interesting is the gene (node) or relationship between two genes (edge). For DoI functions that are built on a different assumption—such as statistical association based on p-values, for which smaller values are more interesting—we transform the values accordingly before loading them into the tool.

Existing approaches that apply the DoI concept to graphs incorporate a diffusion of the DoI over the graph based on a subset of interesting elements—mostly defined by selection [18,19]. In contrast, in our application scenario, we do not have a predefined subset of interesting elements (since the interestingness depends on the task) but instead we have *l* ^′^ DoI values for each graph element, i.e., each node and each edge. To combine and use the DoI functions for filtering, DoI values are required for nodes and edges. As each $\phantom {\dot {i}\!}\text {doi}_{\text {cont}_{l}}$ is based on either a node or edge attribute, we extend the node-based a-priori interest values to edge-related interest values and vice versa. We extend the DoI for vertices $\phantom {\dot {i}\!}\text {doi}_{\text {cont}_{l}}(v_{i})$ to the edges by applying the minimum operator:

$$\text{doi}_{\text{cont}_{l}}(e_{j})=\text{min}\left(\text{doi}_{\text{cont}_{l}}\left(v_{i_{1}}\right),\text{doi}_{\text{cont}_{l}}\left(v_{i_{2}}\right)\right), $$

where $v_{i_{1}}$ and $v_{i_{2}}$ are the two vertices connected by *e*_*j*_. To make sure that an edge remains only visible if both of its vertices are interesting, each edge is assigned the smaller DoI value of the two vertices it is connected to. We extend the DoI for edges $\phantom {\dot {i}\!}\text {doi}_{\text {cont}_{l}}(e_{j})$ to the vertices using the maximum operator:

$$\text{doi}_{\text{cont}_{l}}(v_{i})=\text{max}\left(\text{doi}_{\text{cont}_{l}}(e_{j})\right), $$

∀*e*_*j*_ for which *v*_*i*_ is a source or target. Each vertex is assigned the highest DoI of all edges it is part of, which ensures that a node remains visible as long as any of its edges is visible. The analyst can define a threshold $\theta _{l}\in [0,1],\in \mathbb {R}$ for each $\phantom {\dot {i}\!}\text {doi}_{\text {cont}_{l}}$ function and apply it as a filter to the network.

The discrete DoI functions $\phantom {\dot {i}\!}\text {doi}_{\text {disc}_{k}}\in \{0,1\}$ are derived from the *k* ^′^ knowledge sources $\mathcal {K}_{k}$—a subset of the knowledge sources used to generate the knowledge networks, including, e.g., KEGG, Reactome, and the Gene Ontology (GO-BP, GO-CC, and GO-MF). For each gene *v*_*i*_ that has at least one annotation in $\mathcal {K}_{k}$, e.g., any KEGG pathway from $\mathcal {K}_{\text {KEGG}}$, $\phantom {\dot {i}\!}\text {doi}_{\text {disc}_{k}}(v_{i})=1$, otherwise 0 (if it has no annotation). For each relationship *e*_*j*_ between two genes, the DoI is derived from the two vertices $v_{i_{1}}$ and $v_{i_{2}}$ it connects, where $\phantom {\dot {i}\!}\text {doi}_{\text {disc}_{k}}(e_{j})=1$, if $v_{i_{1}}$ and $v_{i_{2}}$ have at least one annotation in $\mathcal {K}_{k}$ in common, e.g., if they share any KEGG pathway, and $\phantom {\dot {i}\!}\text {doi}_{\text {disc}_{k}}(e_{j})=0$ otherwise.

We allow analysts to interactively define additional continuous DoI functions based on a combination of weighted discrete DoI functions. For this purpose analysts can assign a degree of importance imp_*k*_∈[0,1] to each $\mathcal {K}_{k}$. We normalize these importance values such that $\sum \text {imp}_{k}=1$. The DoI values for nodes *v*_*i*_ of the knowledge-based continuous DoI are derived using the following product:

$$\text{doi}_{\text{cont}_{l}}(v_{i})=1-\prod \left(1-\text{imp}_{k}\right),\,\forall \text{doi}_{\text{disc}_{k}}(v_{i})\neq 0. $$

We use the analogous product to derive the DoI values for the edges *e*_*j*_.

### DoI-based filtering

To support analysts in finding relevant genes (Task I and II), we integrate the concept of DoI-based filtering. In particular, analysts can select a set of continuous DoI functions to dynamically create subsets based on specific conditions in the experimental data (Task I) (see Figure 1(b)). To identify genes that share particular biological properties derived from the integrated knowledge sources (Task II), discrete DoI functions can be used for filtering as well. As both tasks – Task I and Task II – are coupled in Task IV, analysts also need to combine both types of filters. Therefore, *RenoDoI* supports analysts in combining an arbitrary number of a-priori DoI functions to capture interest according to a certain application domain or analysis task.

The discrete DoI functions can be combined using standard first-order logic (∧, ∨) [18,36]:

$$\text{doi}_{\text{disc}} = \left\{ \begin{array}{l l l} \wedge: & \text{doi}_{\text{disc}_{1}}\wedge \text{doi}_{\text{disc}_{2}}\\ \vee: & \text{doi}_{\text{disc}_{1}}\vee \text{doi}_{\text{disc}_{2}} \end{array} \right. $$

In contrast, the combination of continuous DoI functions requires first-order fuzzy logic operations (∧, ∨) [37]:

$${}\text{doi}_{\text{cont}} \,=\, \left\{ \begin{array}{lll} \wedge: & \!\text{doi}_{\text{cont}_{1}}\wedge \text{doi}_{\text{cont}_{2}}\,=\,\text{min}\left(\text{doi}_{\text{cont}_{1}},\,\text{doi}_{\text{cont}_{2}}\right)\\ \vee: & \!\text{doi}_{\text{cont}_{1}}\vee \text{doi}_{\text{cont}_{2}} \,=\, \text{max}(\text{doi}_{\text{cont}_{1}},\,\text{doi}_{\text{cont}_{2}}) \end{array} \right. $$

Before combining the selected continuous DoI functions we apply the user-defined threshold *θ*_*l*_ for each $\phantom {\dot {i}\!}\text {doi}_{\text {cont}_{l}}$, i.e., if $\phantom {\dot {i}\!}\text {doi}_{\text {cont}_{l}}<\theta _{l}$, $\phantom {\dot {i}\!}\text {doi}_{\text {cont}_{l}}=0$. The operators (∧,∨) can be chosen for both function types individually. During our iterative design process and collaboration, it became clear that for the combination of continuous DoI functions the AND operator is preferred, whereas for the combination of discrete DoI functions the OR operator is preferred. Finally, both types of DoI are combined using AND:

$$\text{doi}=\text{min}(\text{doi}_{\text{disc}},\,\text{doi}_{\text{cont}}), $$

i.e., a node (an edge) is only visible if doi_disc_=1, where the resulting fuzzy DoI value (doi) depends on doi_cont_. The resulting fuzzy DoI can be used for filtering, i.e., only elements with doi>0 remain visible.

### Annotation-based layout of subnetworks

**Figure 2:**
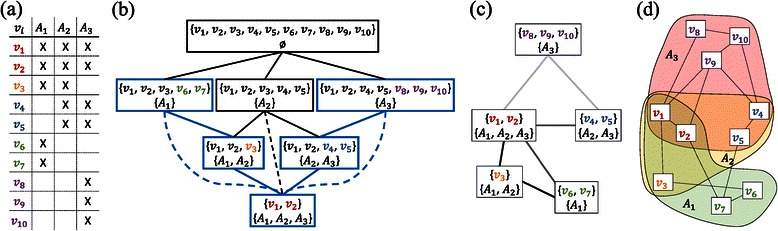
Schematic overview of the layout approach.**(a)** Formal context describing the association of vertices *v*_*i*_ to different annotations *A*_*m*_. **(b)** Result of the formal concept analysis. The final subset graph is derived based on the formal concept, excluding empty subset nodes that occur as we assign vertices only to one subset group (see colored *v*_*i*_’s). Additional edges (dashed links) are added to connect subsets that are more than one hierarchy level apart. **(c)** Our subset graph is laid out using a force-directed layout approach considering edge weights (mapped to shades of gray). **(d)** Each subset node is replaced by the group of vertices it represents, which is laid out independently from the other groups using a force-directed approach.



To support the task of identifying “hub” genes that affect multiple biological processes (Task III) *RenoDoI* allows analysts to semi-automatically create annotation-based layouts for the currently filtered subnetwork (see Figure 1(c)). Our layout approach makes use of the discrete DoI functions (knowledge sources): for each selected $\phantom {\dot {i}\!}\text {doi}_{\text {disc}_{k}}$ function and hence knowledge source $\mathcal {K}_{k}$, we extract the set of annotations from $\mathcal {K}_{k}$ that are associated with any *v*_*i*_ of *G*, with doi(*v*_*i*_)>0, i.e., with any gene that is part of the subnetwork. Hence, we derive a set *A* of annotation groups *A*_*m*_=*V*_*m*_, *V*_*m*_⊆*V*, that overlap, because vertices do often have multiple annotations in each $\mathcal {K}_{k}$. Analysts can choose a set of annotation groups *A* ^′^⊆*A* (see Figure 2(a)) for the subnetwork of interest. The layout of the subnetwork is then computed with a two-step layout approach based on the selected annotation groups *A*_*m*_∈*A* ^′^ as described in Algorithm 1.

Since we want to visualize the different intersection groups explicitly, we developed an approach similar to that of Itoh et al. [22]; however, our approach is based on a different subset graph (meta-graph) and uses a different layout within the intersection groups. We derive the subset graph *G* ^subset^ from the selected annotation groups *A*_*m*_ by applying a formal concept analysis [38] (see Figure 2(a) and (b)). The nodes of the resulting subset graph represent non-empty sets and intersections of them (see Figures 2(c)). Before we compute the layout with the force-based model by Fruchterman and Reingold [39], we assign edge weights to each of the edges in *G* ^subset^, which are indirectly proportional to the size of the two subset nodes $v_{n}^{\text {subset}}$ they connect (in Figure 2(c), these edge weights are mapped to shades of gray).

We achieve our goal of positioning “hub” genes in-between the groups they are associated with, since our subset graphs are sparse, i.e., they represent a subgraph of the concept lattice graph. We visualize the subset graph as an intermediate result to provide analysts the opportunity to adjust the layout to their needs. Once the analyst is satisfied with the layout of the subset graph, each group of nodes, i.e., all $v_{i}\in v_{n}^{\text {subset}}$, is laid out individually within an area around the position of the respective subset node $v_{n}^{\text {subset}}$ using a force-directed layout (see Figure 2(d)).

### Comparing subnetworks

*RenoDoI* also provides a feature for comparing subnetworks derived by using different sets of annotation groups *A* ^′^ based on different or the same discrete DoI functions $\phantom {\dot {i}\!}\text {doi}_{\text {disc}_{k}}$ (Task V). A set of subnetworks could be compared using the available subnetwork views in Cytoscape by arranging them next to each other (juxtaposition) and using explicit encoding based on coordinated multiple views brushing (the selection of nodes in one subnetwork will highlight these nodes in all other subnetworks in which they are present). However, using simple juxtaposition, similarities and differences are hard to capture.

Therefore, we use the superposition concept instead and provide the analyst with an additional network view showing the super-graph of the to be compared subnetworks (see Figure 1(d)). Since we want to support the analyst in maintaining the mental map, we need to minimize layout changes in the super-graph compared to the underlying subnetworks. To achieve this goal, we compute a zlayout using the largest subnetwork as a reference. We first initialize the super-graph as a copy of the reference subset graph *G* ^subset^. Next, any group (subset node ${{v_{n}^{\text {subset}}}'}$) that is not yet part of the reference subset graph is added to the super-graph *G* ^super^. Every ${v_{n}^{\text {subset}}}'$ will thereby be positioned close to those—already existing—subsets ${v_{n}^{\text {subset}}}$ it overlaps with, preferably those with high overlap or with a high number of topological connections. Therefore, any ${v_{n}^{\text {subset}}}'$ is connected with at least one or more subsets $v_{n}^{\text {subset}}$ it overlaps with, where the edge weights depend on the size of the overlap $\left (\left |{v_{n}^{\text {subset}}}' \cap v_{n}^{\text {subset}}\right |\right)$ or—in case of similar sizes of overlaps—the number of edges connecting vertices *v*_*i*_ from the added subset ${v_{n}^{\text {subset}}}'$ with vertices from the overlapping subsets $v_{n}^{\text {subset}}$. The super-graph is then laid out using the same edge weighting and layout approach as we used for the individual subset graphs, with the restriction that only the added nodes ${v_{n}^{\text {subset}}}'$ (the ones that are not part of the reference subset graph) are allowed to change their position. Similar to the two-step layout approach for individual subnetworks, in the second step, each group of nodes (all *v*_*i*_ associated with a ${v_{n}^{\text {subset}}}'$) is laid out individually, where only the node groups of the added subsets ${v_{n}^{\text {subset}}}'$ are laid out.

### Analysis of experimental data

**Figure 3:**
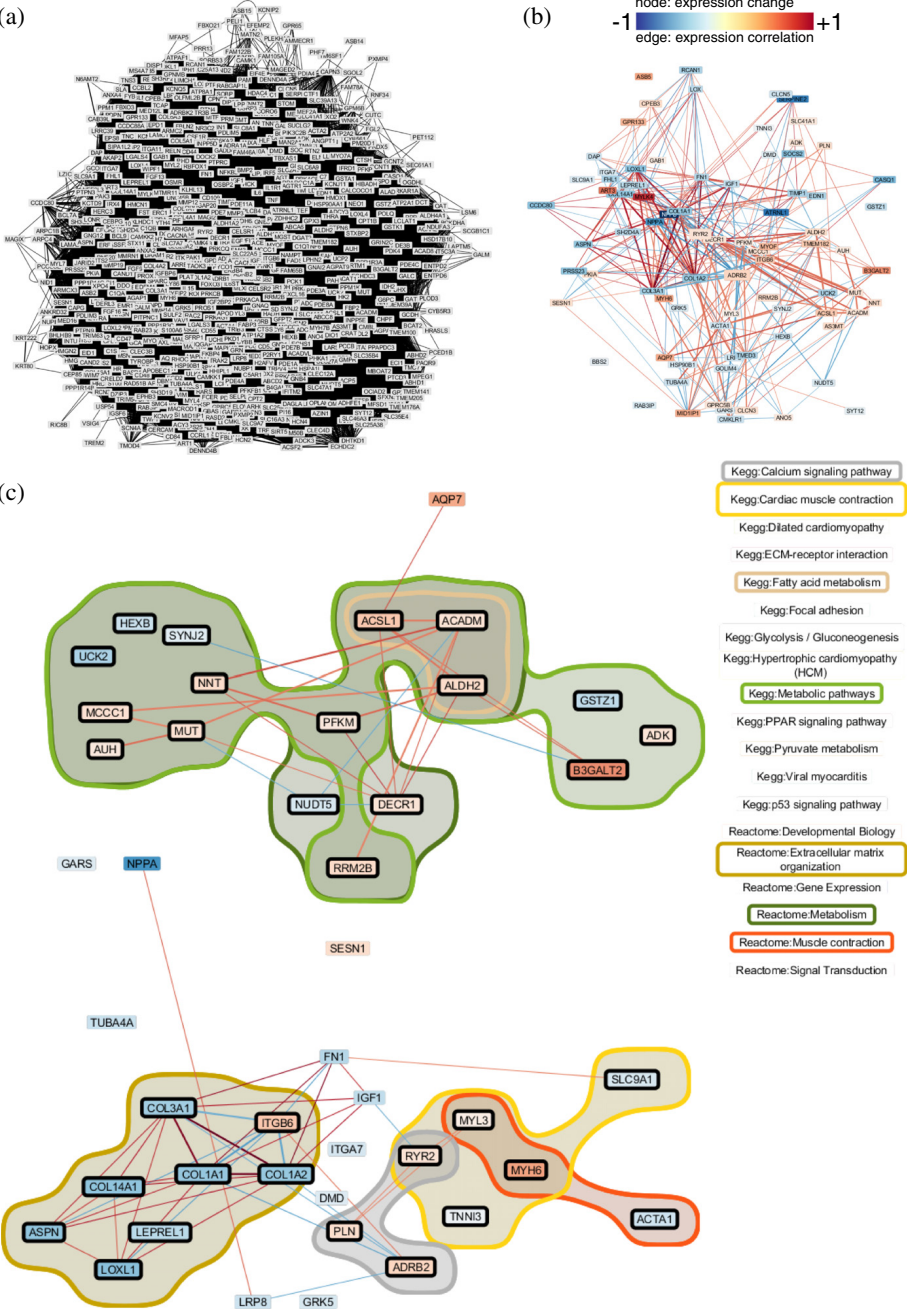
Comparing myocardial gene expression findings with components of the *β*1-adrenergic signaling network.**(a)** The integrated data-knowledge network comprising 711 genes and 74,373 relationships between them. **(b)** Filtered subnetwork after applying two continuous DoI functions. In particular, the analyst used the statistical association of gene expression with LVEF response and the gene-expression correlation as DoI functions. In addition, the difference in the magnitude of change in gene expression was then mapped to the color of nodes and the gene expression correlation to the color of edges. **(c)** Further filtering and layout of the subnetwork in **(b)** based on two discrete DoI functions of interest: KEGG and Reactome. The subnetwork was laid out based on KEGG and Reactome pathways that are involved in heart function. Annotations related to metabolism, extracellular matrix organization, calcium signaling, or muscle contraction are selected to highlight genes associated with them.

**Figure 4:**
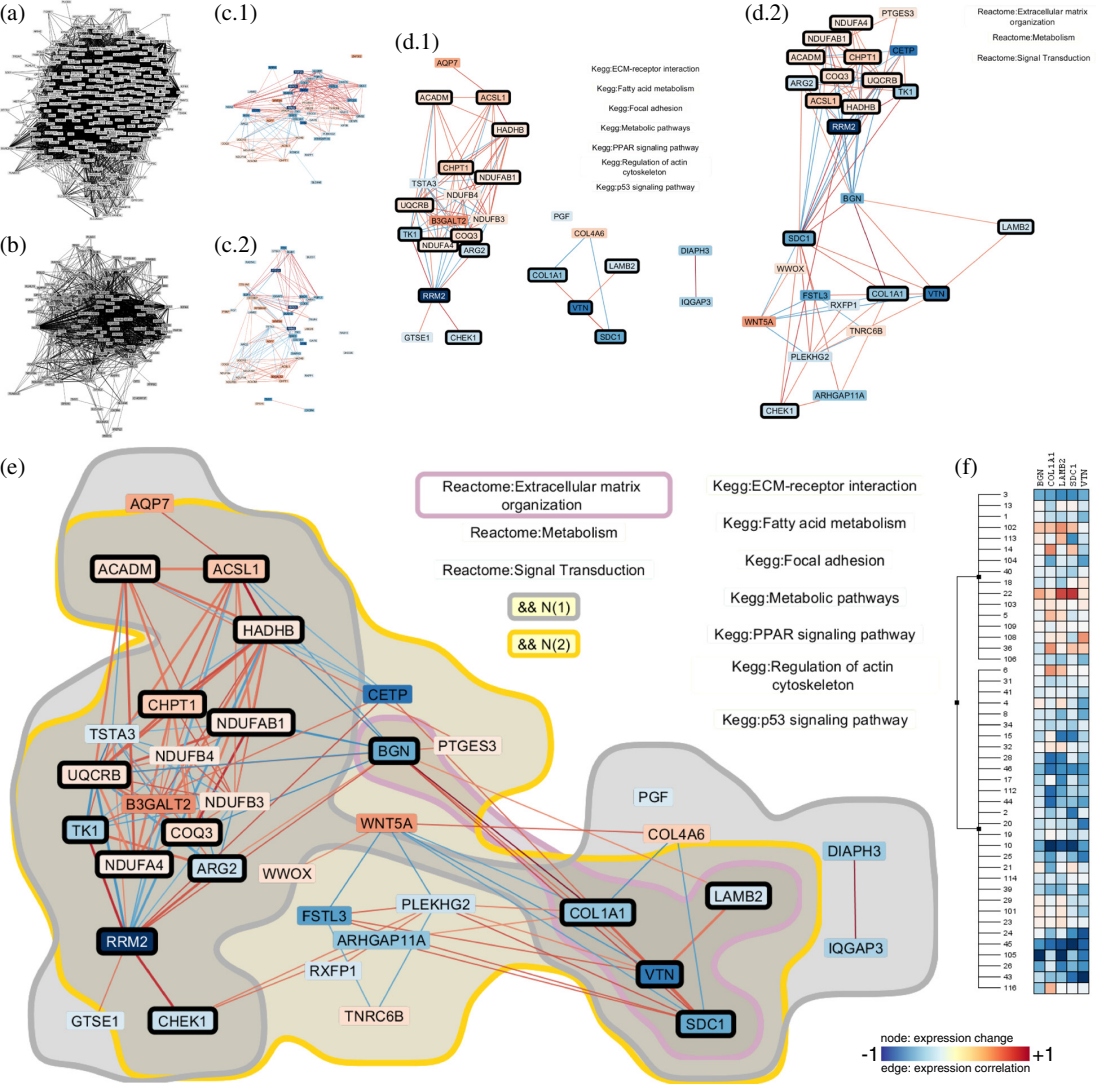
Integrating and comparing knowledge from multiple sources to explore novel findings in experimental data.**(a)** The complete data-knowledge network. **(b)** Filtered subnetwork after applying two continuous DoI functions. Again, the analyst used the statistical association of gene expression with LVEF response and the gene-expression correlation as DoI functions. Next, two discrete DoI functions are applied to extract subnetworks based on different knowledge sources **(c.1)** and **(c.2)**. Individual subnetworks based on similar terms from KEGG **(d.1)** and Reactome **(d.2)** were created to analyze gene annotations individually. **(e)** Integrated data-knowledge network for comparison and analysis of the overlap. Genes contained in both subnetworks are highlighted by the black border (in **(d.1)**, **(d.2)**, and **(e)**) and both sets of genes—one set for each network—are surrounded by a contour. The analyst determined that the expression of multiple extracellular matrix proteins—highlighted and surrounded by a contour—according to both expert sources were correlated with LVEF response—confirmed using the heat-map view **(f)**. In **(c)**-**(e)**, the same color mapping is applied as in Figure 3.

**Figure 5:**
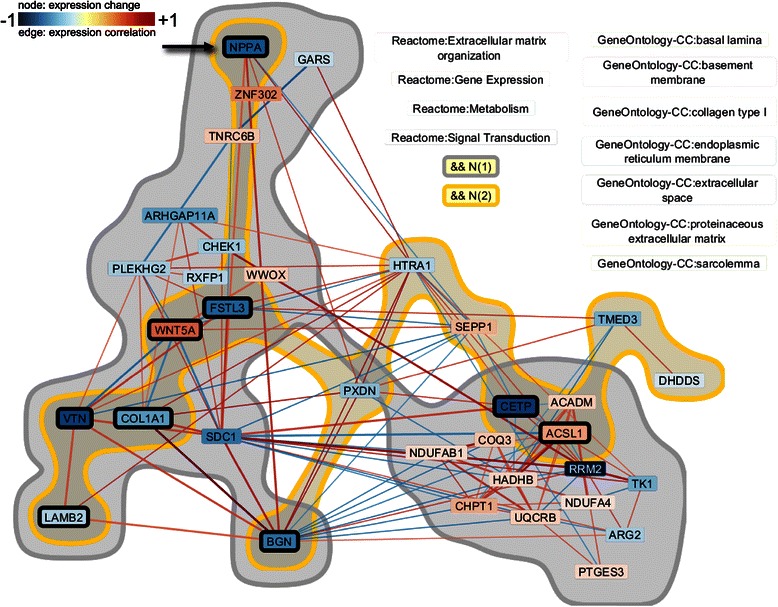
Integrating and comparing knowledge from multiple sources to explore novel findings in experimental data: Combined network of the derived subnetworks using the experts Gene-Ontology Cellular Compartment and Reactome. Genes contained in both subnetworks are highlighted by the black border to investigate the overlap between these two experts. NPPA is one of the genes with extracellular products we identified that might be used as diagnostics in peripheral circulation. The same color mapping as in Figures 3 and 4 was applied.

Once, genes and biological relationships among them that are associated with the phenotype of interest have been identified, the analyst moves on to investigate the transcriptomic profiling data for these genes. To understand the molecular mechanism of clinical response to a treatment, it is necessary to investigate the experimental data used to generate the data-based relationships, i.e., the transcriptomic profiling data (Task II and IV). This involves, e.g., the investigation of statistical measures such as the gene-expression correlation or the difference in gene expression between conditions. *RenoDoI* allows analysts to map one of the available numerical attribute of the genes (or the relationships between them) to the color of nodes or edges (see Figures 3, 4 and 5). As most of these attributes include negative as well as positive values, which—according to our biomedical collaborators—should be easily distinguishable, we selected a diverging color scale from ColorBrewer [40]. *RenoDoI* allows analysts not only to investigate the statistical summaries of the experimental data but also the individual transcriptomic profiling data, e.g., details for individual patients. *RenoDoI* integrates the functionality to open a heat-map view for a set of selected genes, where rows represent conditions, such as patients or treatments, and columns represent genes.

### Interaction

Each subnetwork view includes an interactive legend that shows all annotation sets *A*_*m*_∈*A* ^′^, each labeled with its name and discrete DoI function $\phantom {\dot {i}\!}\text {doi}_{\text {disc}_{k}}$, which corresponds to the knowledge source $\mathcal {K}_{k}$ to which it belongs. For the super-graph, the legend additionally includes a network label for each of the subnetworks included in the network comparison the super-graph represents.

The legend is linked to the main-network and all subnetworks via brushing and linking such that the selection of any *A*_*m*_ within the legend results in the highlighting of all associated nodes (genes) *v*_*i*_∈*V*_*m*_, *V*_*m*_⊆*V*, in these network views (see Figures 3 and 4). The subnetwork labels can be used to highlight all nodes of the respective subnetwork or—in case two or more such labels are selected—the intersection between these networks (see Figure 5). Nodes are highlighted by drawing them slightly bigger and by adding a thick black border. (We cannot use the default feature of yellow highlighting for nodes since we already map other attributes to the node color.) To visualize the set memberships each selected set of nodes will be surrounded by an isocontour based on the BubbleSets approach [27]. We decided against using convex hulls or Euler diagrams, as these types of contours may include nodes that are actually not associated with the underlying group and therefore could lead to confusion.

By selecting a node *v*_*i*_ within a subnetwork, each annotation set *A*_*m*_ or network label it is associated with (i.e., each *A*_*m*_ with *v*_*i*_∈*A*_*m*_) will be highlighted in the legend of the respective subnetwork view and the respective isocontours will be shown. Each annotation and hence isocontour is assigned a color based on a qualitative colormap created with ColorBrewer [40]. The heat-map views are also linked to the network views in such a way that the selection of one or more columns and hence genes in the heat-map will highlight the respective nodes in the networks and vice versa.

## Results

In this section we describe in two case studies how *RenoDoI* was used by a biomedical researcher to analyze a complex dataset that combines clinical data with high-throughput data. We first describe the biomedical goals of the studies and provide the details of the underlying dataset; following we describe the high-level workflow for the analytical scenarios. A more detailed description of the stepwise workflow of both case studies is included in the supplemental material. The accompanying video—a screencast of the interactive analysis of the heart failure study using *RenoDoI*—illustrates the interactive features of our system (http://youtu.be/yeJaSYkA0-Q).

### Biomedical goals

Heart failure (HF) is the leading cause of hospitalization in US patients age >65, and approximately 50% of patients die within 5 years of diagnosis despite great advances in treatment over the past 15 years [41]. *β*-blocker therapy has been shown repeatedly to improve outcomes, but only 60–70% of patients with systolic HF demonstrate improvement. High-throughput gene expression profiling may provide additional insight into the mechanisms of *β*-blocker response and possibly identify diagnostic candidates to predict *β*-blocker response. We have worked with a group of cardiologists who obtained biopsies of heart tissue from HF patients as they started on *β*-adrenergic receptor blockers, a standard heart failure therapy. Kao et al. [42] describe the details of the clinical trial, its study design, and the results from a statistical analysis. Through weekly meetings over the course of several months, we identified three primary analysis goals in collaboration with one of the domain experts.

**Understand the molecular mechanism of clinical response to** ***β*** **-blocker therapy** The specific targets of *β*-blockers are well described, and the effectiveness of *β*-blockers in HF is proven. However, the mechanism by which *β*-adrenergic receptor blockade results in improved myocardial contraction is incompletely understood. Better understanding of the molecular mechanisms of *β*-blocker response may allow improved prediction of likelihood of response and identify potential new therapeutic targets for HF.

**Understand pathological molecular changes associated with HF** HF is associated with reversion of expression of several signaling and contractile genes to a fetal gene program. However, only a small number of genes have been characterized in this manner. Whole transcriptomic analysis could greatly expand the number of genes whose expression levels change with HF, potentially allowing improved insight into the determinants of these changes.

**Identify medical interventions that may increase the likelihood of** ***β*** **-blocker response in HF**. Approximately one-third of HF patients who receive *β*-blocker treatment do not show improvement in cardiac function. However, the molecular determinants of individual response to *β*-blockers are not known. Based on a comprehensive analysis of gene expression changes, it may be possible to identify determinants of *β*-blocker response amenable to medical therapy. If so, it may be possible to augment or increase the likelihood of *β*-blocker response.

### Biomedical data

Our data includes clinical data and transcriptomic profiling data and knowledge-based data. *Clinical data* refers to tests performed on HF patients during the study. For this analysis, the most relevant elements include presence of HF, assignment to one of three *β*-blocker treatment arms, and left ventricular ejection fraction (LVEF) measured at baseline, 3 and 12 months following initiation of *β*-blockers. LVEF response was defined as an increase from baseline of ≥8 units at 12 months or if no 12 month value was available, ≥5 units at 3 months. This study was conducted between 2000 and 2008 at the Universities of Colorado and Utah in accordance with the Declaration of Helsinki and included a Data Safety Monitoring Board. The study was approved by institutional review boards at both sites (Colorado Multiple Institutional Review Board protocol 00-242), and all subjects gave written informed consent.

*Transcriptomic profiling data* refers to longitudinal measurement of myocardial gene expression in HF patients during the study. Gene expression is measured by quantifying mRNA fragments called transcripts that encode proteins. Changing the quantity of mRNA fragments is one component of regulating the amount of a given protein is produced. In the case of HF, it has been previously shown that transcript levels of certain genes in HF patients more closely resemble levels in the developing heart prior to birth (‘fetal gene program’) than normal adult heart tissue. Furthermore, transcript levels of these genes return to an adult pattern when heart function improves as a result of *β*-blocker therapy. Enrolled patients had myocardial biopsies performed at baseline, 3 and 12 months in coordination with LVEF measurements. Gene transcript levels were quantified using the Affymetrix HGU-133 Plus 2 microarray. Differences in gene expression between LVEF responders and non-responders were tested using the R statistical package and SAS.

Transcriptomic profiling data may be compared between groups of clinically distinct patients, in this case between LVEF responders and non-responders, in order to identify potential mechanisms of positive drug response. Integrating signaling pathway and molecular function knowledge can enrich these comparisons by identifying groups of genes with known biological relationships that are relevant to the phenotype of improved cardiac function.

### Comparing myocardial gene expression findings with components of the *β*1-adrenergic signaling network

As our analyst was specifically interested in comparing experimental findings with known components of the *β*1-adrenergic signaling network, he selected 711 genes for analysis based on prior work suggesting a relationship between *β*1-AR activity and gene expression or *β*1-AR signal transduction. (These genes were identified by measuring myocardial gene expression in transgenic mice over-expressing *β*11-Arg389 and *β*11-Gly389 adrenergic receptors compared with littermate control animals.) An integrated data-knowledge network comprising these 711 genes and 74,373 relationships between them (see Figure 3(a)) was constructed as described in the data model section. We used 26 different knowledge sources and derived 10 discrete DoI functions. A detailed description and illustration of the analysis steps for the first case study are available as Additional file 1.

In order to focus on findings relevant to the primary analytical question, the analyst first focused on the visualization of relevant experimental data (Task I). He reduced the complexity of the data by filtering genes whose expression is associated with the phenotype—here left ventricular ejection fraction (LVEF) response. In particular, he used the statistical association of gene expression with LVEF response as a DoI function (the corresponding p-values were transformed as described in the Methods Section to match the semantic meaning of our DoI functions) that permitted dynamic filtering of nodes with low p-value. He started with a very strict threshold (a p-value of <0.01 for expression change in responders compared with non-responders was considered significant) and then iteratively relaxed it until the filtered network had a reasonable size (p-value of ≤0.05). To focus on genes with evidence of meaningful correlation, he added a second DoI function allowing dynamic filtering of high gene-gene correlation values (using a threshold *θ*≥0.5). Both DoI functions are based on the measures of the last observation carried forward. In addition, the difference in the magnitude of change in gene expression was then mapped to the color of nodes and the gene expression correlation to the color of edges (see Figure 3(b)), respectively.

To explore which biological processes and pathways may be associated with *β*1-adrenergic signaling-related genes and LVEF response (Task II), the analyst created a subnetwork based on shared gene properties. In particular, he selected specific knowledge sources (discrete DoI functions) of interest—KEGG and Reactome—and created an intermediate subnetwork from visible genes based on shared annotations from these knowledge sources only. To identify “hub” genes (Task III) he then rearranged the genes of this subnetwork of individual genes to reflect the existing knowledge of the filtered genes and their relationships in myocardial contractile function. In particular, he used the annotation- and knowledge-driven layout approach to generate a layout of the subnetwork based on KEGG and Reactome pathways that are involved in heart function (see Figure 3(c)), e.g., muscle contraction and calcium processing.

Based on this layout and the evidence of change in a number of metabolic genes and extracellular matrix proteins, the analyst could identify several genes implicated in the *β*1-AR network that have been suspected to be relevant to LVEF improvement, but were not previously characterized. In combination with the color mapping, he could conduct a rapid survey of coordinated and correlated expression changes in various pathways (Task IV). Correlated up-regulation of contractile and calcium handling genes is apparent, although there is also clear coordinated up-regulation of several metabolic genes and correlated down-regulation of extra-cellular matrix proteins, which is consistent with improved myocardial efficiency and slowing of pathologic remodeling. The analyst indicated that these findings in particular and the ability to explore these data in the context of specific knowledge sources will provide hypotheses for future confirmatory clinical studies regarding diagnostics and the likelihood of LVEF response to *β*-blockers as well as possible interventions to increase the effectiveness of *β*-blockers.

### Integrate and compare knowledge from expert sources to explore novel findings in experimental data

Multiple statistical analyses identified expression of a cholesterol modifying protein (CETP) as being strongly associated with LVEF response [43]. Not only was cholesterol trafficking not expected to have a role in recovery of heart function, but the gene product is the target of a new class of cholesterol-lowering medications currently in development, raising the possibility that these new drugs may impact heart failure. Therefore, the analyst explored the plausibility and possible mechanisms of this novel hypothesis using an integrated data-knowledge network comprising 328 genes and 11,081 relationships between them (see Figure 4(a)). We used again 26 different knowledge sources and derived 10 discrete DoI functions. This network was composed of genes whose expression was most highly correlated with CETP in all patients at all time points. Also for the second case study, a detailed description and illustration of the analysis steps is available as Additional file 2.

For the analysis of this network, the analyst used the same two DoI functions as before: first, he used the statistical association of gene expression with LVEF response as a DoI function with a p-value of ≤0.05 and, second, the gene expression correlation using a threshold *θ*_2_≥0.5 (see Figure 4(b)). Again, he mapped the difference in the magnitude of change in gene expression to the color of nodes and the gene expression correlation to the color of edges (in Figures 4(c)-(e) and 5), respectively.

In the first phase, the analyst wanted to identify pathways associated with genes whose expression is correlated with CETP (Task II). Because essentially nothing is known about CETP in heart failure, he identified genes with consistent pathway annotations. He created individual subnetworks based on similar terms from Reactome and KEGG (Task III; see Figures 4(d.1) and (d.2)). He then created a combined network (see Figure 4(e)) and dynamically viewed gene annotations from each expert individually, in union, and the intersection between the two networks (Task V; see Figures 4(d.1), (d.2), and (e)). Based on these operations he determined that expression of multiple extracellular matrix proteins and metabolic proteins according to both expert sources were correlated with LVEF response (see Figure 4(e)) and confirmed this finding using the heat-map view (see Figure 4(f)).

In order to explore relationships between correlated genes along multiple axes the analyst combined another two subnetworks (Task V). In particular, he visualized both the cellular localization of each gene as well as their functional/pathway annotations by creating subnetworks using the Gene-Ontology Cellular Compartment expert and the Reactome functional annotations (Task III; see Figure 5). By exploring the overlap between these two experts in a combined network, he found clusters of correlated genes within specific compartments, within specific pathways, and functionally related genes localized to the same compartment. These findings can (a) support confirmatory translational experiments by identifying specific candidate genes in specific cellular compartments to isolate and (b) identify genes with extracellular products (such as NPPA) that might be used as diagnostics in peripheral circulation. Incorporation of pharmaceutical target experts would also allow the identification of candidate therapeutic targets to support drug repurposing or novel drug application development.

## Discussion

Our visual analytics approach implemented in *RenoDoI* supports the investigation of large and dense integrated data-knowledge networks; until now, these types of analyses were time-consuming and costly since it was not possible to perform all the steps necessary for the analysis in an efficient and integrated workflow. We now discuss our development process, limitations of the tool, and its generalizability.

### Iterative design and development process

We developed *RenoDoI* through an iterative design process using formative evaluation that bears resemblance to the pair analytics approach [44]; we closely collaborated with a group of biomedical researchers from the Division of Cardiology in the CU Medical School. Due to our regular meetings—typically on a weekly basis—we were able to develop a deep understanding of the analysis needs and to include regular and early feedback during the development process. In comparison to Hanalyzer and other knowledge-based approaches, *RenoDoI* is much more flexible and supports a data-driven as well as knowledge-driven analysis approach. *RenoDoI* supports the interactive and fast extraction of relevant subnetworks based on DoI functions within seconds or at most minutes, provided that suitable DoI functions representing the analyst’s interest are available.

### Tool limitations

*RenoDoI* still has some limitations that we plan to address in the future. Although, compared to Hanalyzer, it improves the analytical flexibility with respect to the size of the network, scalability can still be further improved. Ideally, analysts would like to extract middle-sized networks with hundreds of nodes—as analyzed in our case studies—from the complete dataset (in our dataset are about 20,000 genes) directly within *RenoDoI* using additional knowledge or data sources but independent from its visualization. This is not possible with the current implementation. We tested *RenoDoI* on a larger network with more than 2,600 nodes and 600,000 edges on a standard desktop computer with 16GB of memory. While the computational speed for applying DoI functions was still acceptable (in the range of 2 to 3 seconds), the rendering of the resulting networks could take between 7 and 50 seconds (depending on the network size and whether graphics details are rendered) and therefore no longer supports an interactive workflow. The integration of additional statistical methods could further improve the overall utility of *RenoDoI*. It would support analysts not only in hypothesis generation but also creating publishable statistical results to enhance the more qualitative findings from the network analysis. Finally, a feature for network subtraction could complement the network comparison feature and allow analysts to study the differences in networks representing different patient groups, e.g., female vs. male patients.

### Generalizability

Even though we derived the analytical tasks from a selected use case, they are not bound to a specific dataset but are generalizable to a large set of knowledge-based analyses. *RenoDoI* can be applied to any experimental data that produces measurements on genes, gene products, or for which the measurements can be mapped onto genes as it was done in previous applications of the Hanalyzer [2-4]. Our collaborators are already analyzing additional networks constructed to investigate the role of the HOX gene family, previously unstudied in heart failure, and gene expression variation associated with adrenergic receptor genotypes. We recently also began a new collaboration in which we use *RenoDoI* to analyze and explore proteomics data in the context of spinal coord injury.

## Conclusion

Scalability is often a serious bottleneck for visualization approaches in the context of big data; genome scale data is certainly no exception. Especially in molecular biology, there is a surfeit of available information, and visualizing all of it at once reduces the value of a visualization or makes it even unusable for analytical purposes. The overwhelming nature of many contemporary biological network visualization approaches has led to the widespread recognition of the problems of understanding or even just navigating “hairballs”. Therefore, a critical problem is the need to reduce the complexity of displayed information by prioritizing and displaying only what is important to the analyst for solving a specific task. We addressed this problem by integrating DoI functions for filtering with graph layouts based on group associations, and a network comparison technique, to support analysts in untangling dense biological networks.

*RenoDoI* is the first approach that supports the integrated visualization and analysis of both existing knowledge (from databases and the literature) and experimental data in a dynamic, task-driven, customizable fashion. We demonstrated the utility of *RenoDoI* in two case studies using a large and complex dataset from a clinical trial that contains clinical data as well as transcriptomic profiling data. These types of datasets reflect current data analysis needs from multiple areas, including translational research, systems biology, and molecular biology. Our collaborators are excited about the analytical capabilities of *RenoDoI* and believe it can significantly enrich and speed-up the analysis of their data.

## Availability and requirements

*RenoDoI* is implemented in the Java™programming language as an application in Cytoscape [6]. It is available in the Cytoscape App Store http://apps.cytoscape.org/apps/renodoi as a free component, along with a manual and a demo video. It requires Cytoscape version 3.2 or later to run.

## Additional files

Additional file 1
**Case Study 1.** Detailed description of the analysis workflow of the first case study: Compare myocardial gene expression findings with components of the *β*1-adrenergic signaling network.

Additional file 2
**Case Study 2.** Detailed description of the analysis workflow of the second case study: Integrate and compare knowledge from expert sources to explore novel findings in experimental data.

## Abbreviations

DoI: Degree-of-interest
GAD: Genetic association database
GO: Gene ontology
HF: Heart failure
LVEF: Left ventricular ejection fraction
