# Supplementary material for: Visual analysis of biological data-knowledge networks
Source: BMC Bioinformatics. 2015 Apr 29;16:135. doi: 10.1186/s12859-015-0550-z (PMC4456720; doi:10.1186/s12859-015-0550-z)

Case Study 1

Comparing myocardial gene expression findings with components of the β-adrenergic signaling network

To elucidate possible mechanisms by which β-blocker therapy results in recovery of left ventricular systolic function, we used the previously described longitudinal myocardial gene expression data from HF patients.

LVEF response status, baseline and follow-up gene expression values and gene-gene correlation values for 19,620 genes were available for 47 patients.

As we were specifically interested in comparing experimental findings with known components of the β-adrenergic signaling network, 711 genes were selected for analysis based on prior work suggesting a relationship between β-AR activity and gene expression or β-AR signal transduction.

A combined data-knowledge network comprising these 711 genes and 74373 relations between them was constructed.

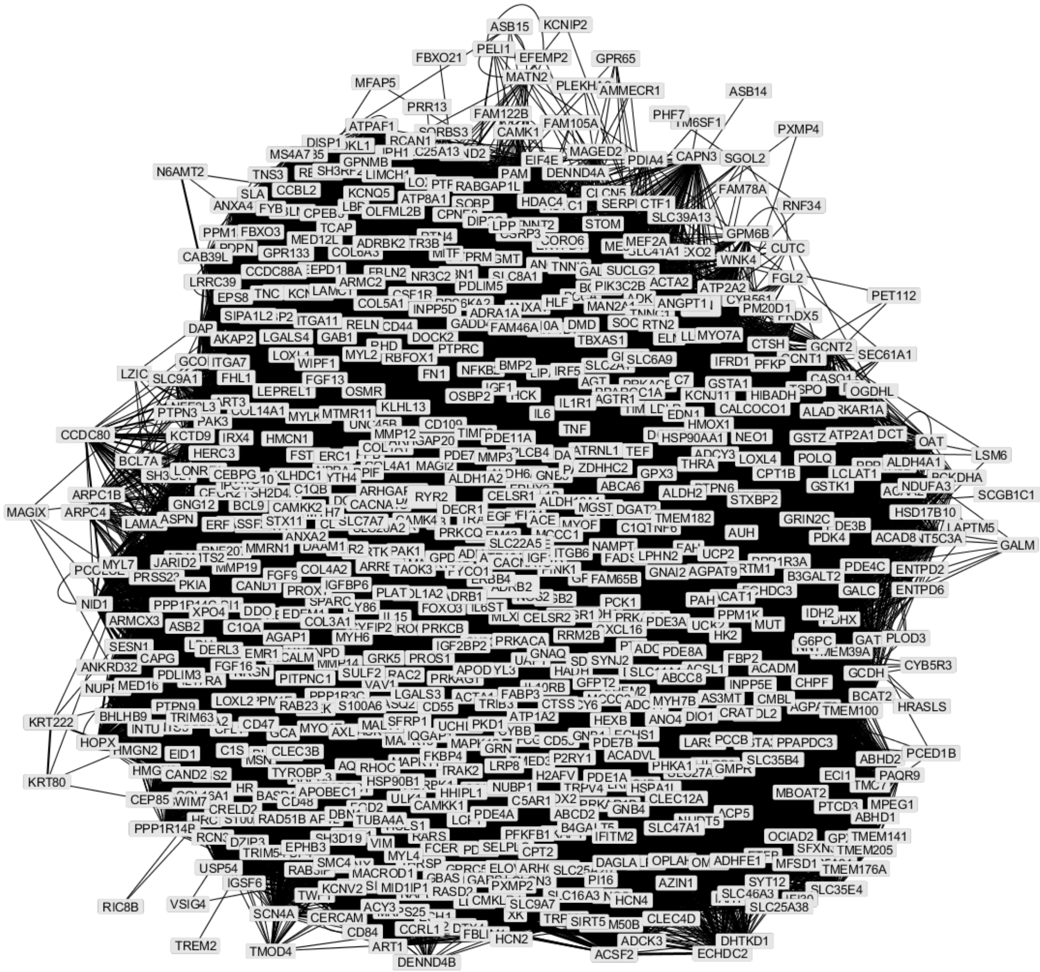

The combined data-knowledge network comprising 711 genes and 74373 relations between them.

Step2 - Filtering based on continuous DoI functions based on the experimental data: In order to focus on findings relevant to the primary analytical question, we first focused on the visualization of relevant experimental data. Therefore, we reduced the complexity of the data by filtering genes whose expression is associated with the phenotype – here LVEF response. In particular, we used the inverted statistical association of gene expression with LVEF response as DoI function – inverted as small p-values are of interest – that permitted dynamic filtering of nodes with low p-value (p-value<0.05). This way we could filter genes with a statistical difference in correlation between responders and non-responders. To focus on genes with evidence of meaningful correlation, we added a second DoI function allowing dynamic filtering of high gene-gene correlation values (|correlation|>0.5).

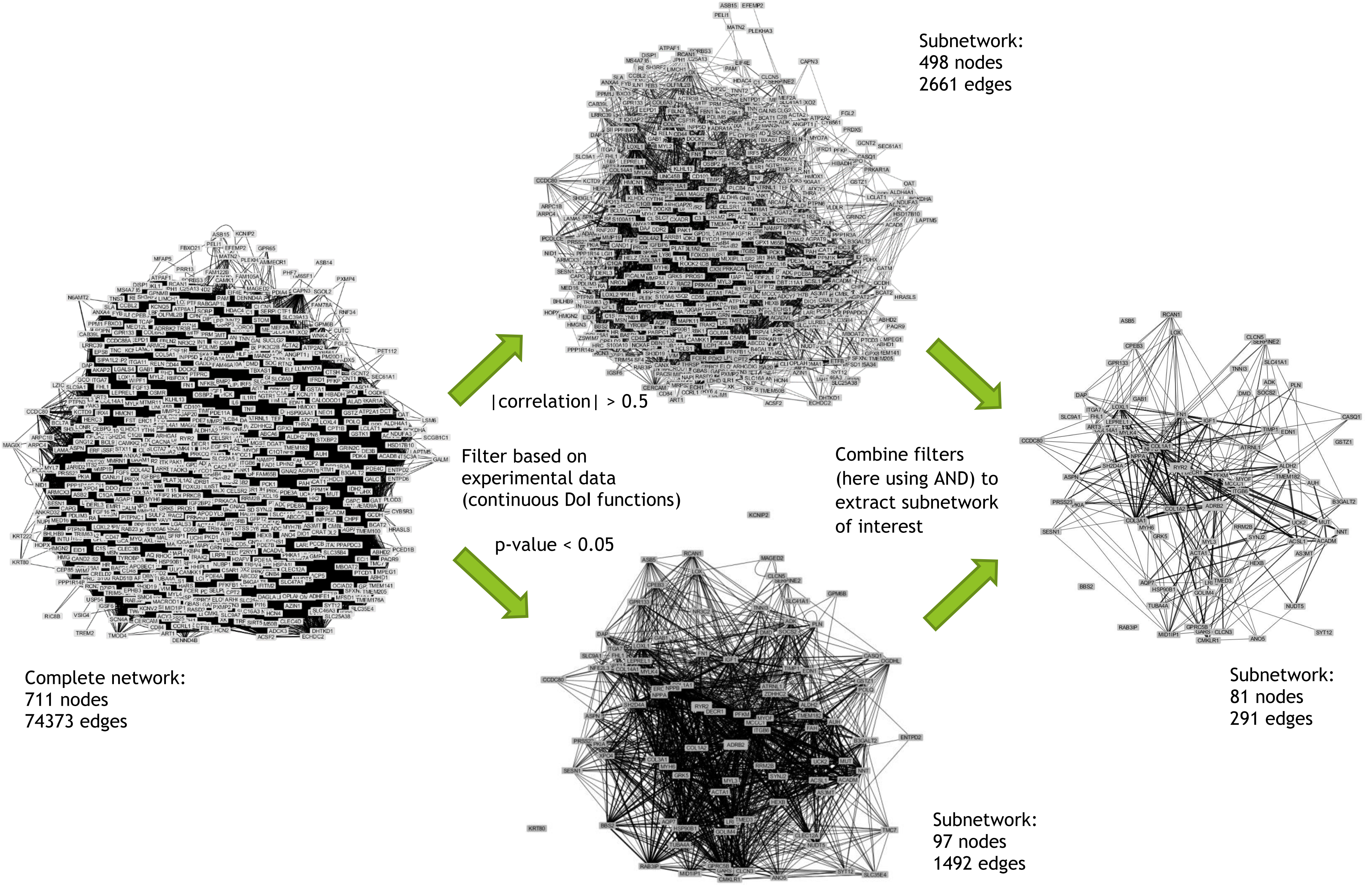

Step2 – Mapping of experimental data: The magnitude of change in gene expression and correlation was then mapped to the color of nodes and edges, respectively. Significantly up-regulated (down-regulated) genes show up as highly saturated red (blue) nodes. Gene expression correlation is mapped to color using the same color map, i.e., high positive (negative) correlations are mapped to a highly saturated red (blue).

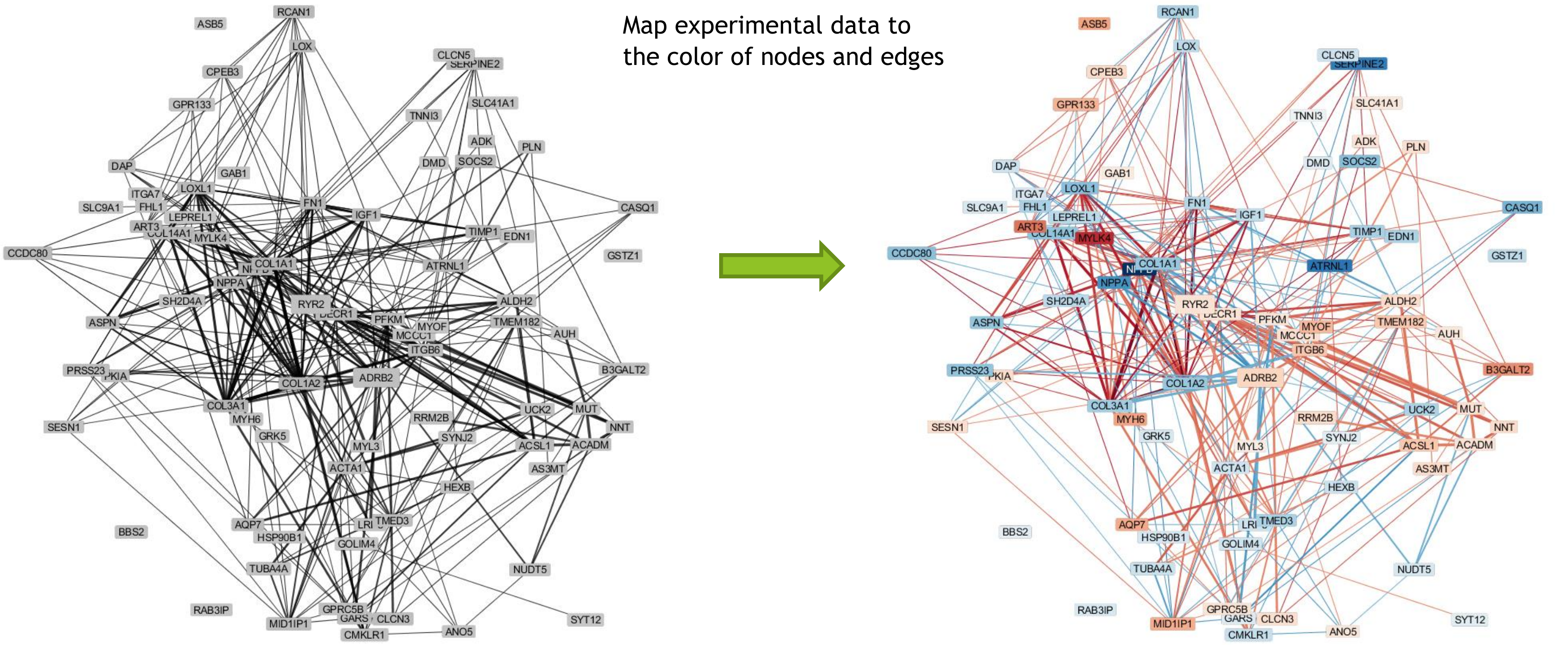

Step 3 – Filtering based on discrete DoI functions based on knowledge sources: To explore which biological processes and pathways may be associated with ß-adrenergic signaling-related genes associated and LVEF response, a subnetwork based on shared gene properties was created. In particular, we selected two knowledge sources (discrete DoI functions) of interest - KEGG and Reactome - and created an intermediate subnetwork from visible genes based on shared annotations from these knowledge sources only.

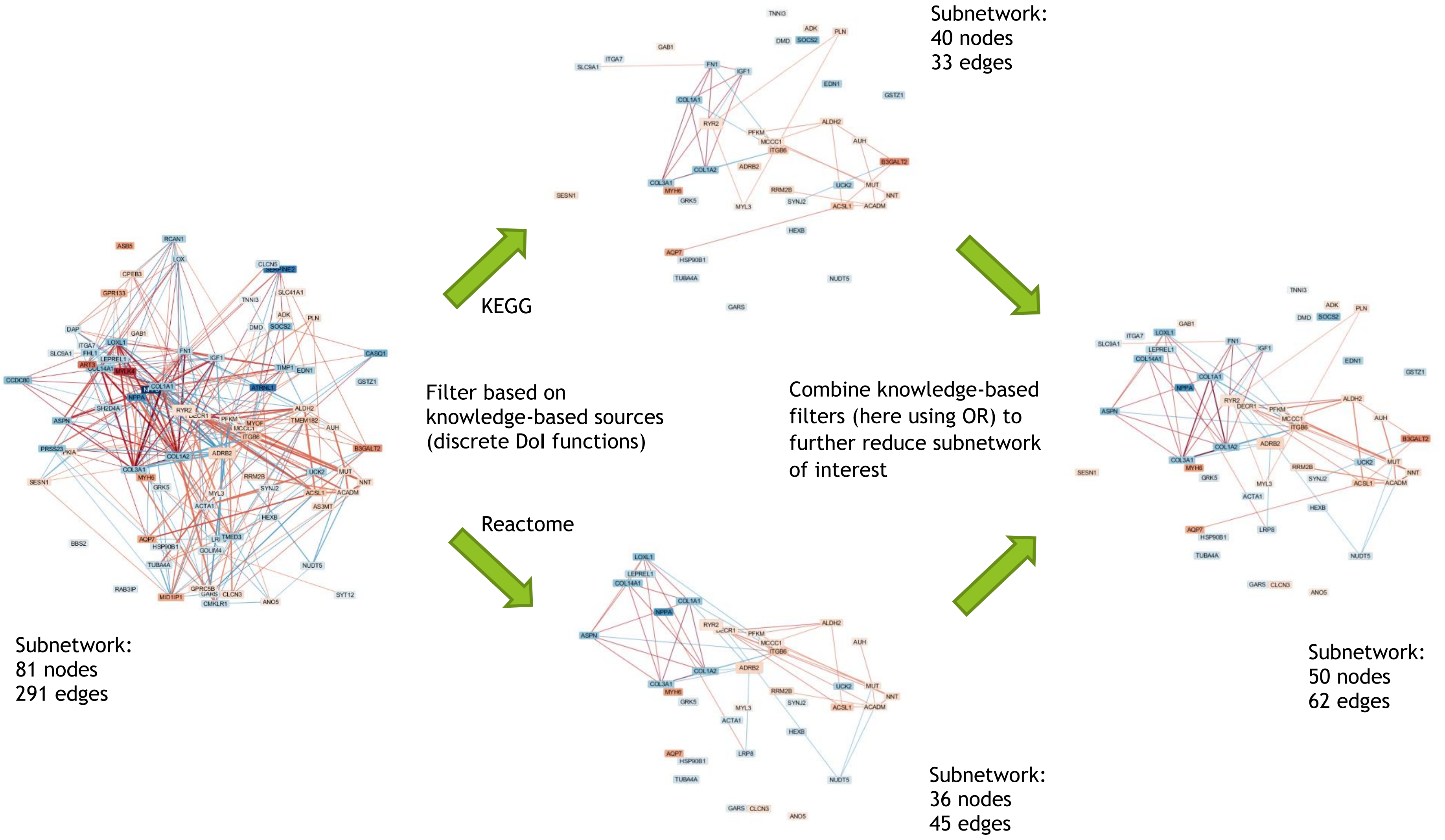

Step 4 - Annotation-based layout of subnetwork: We rearranged the genes of the subnetwork of individual genes to reflect to existing knowledge of the filtered genes and their relationships in myocardial contractile function. In particular, we generated a laid out the subnetwork based on KEGG and Reactome pathways that are involved in heart function using the annotation- and knowledge-driven layout approach. The KEGG pathways include: calcium signaling pathway, cardiac muscle contraction, dilated cardiomyopathy, ECM-receptor interaction, fatty acid metabolism, focal adhesion, glycolysis/gluconeogenesis, hypertrophic cardiomyopathy (HCM), metabolic pathways, PPAR signaling pathway, pyruvate metabolism, viral myocarditis, and p53 signaling pathway. The Reactome pathways include: developmental biology, extracellular matrix organization, gene expression, metabolism, muscle contraction, and signal transduction.

Filtered subnetwork

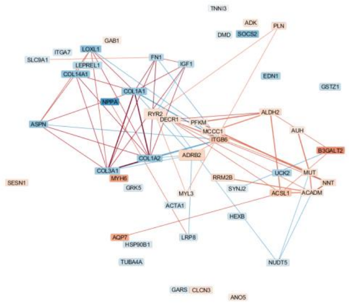

Layout of the subset-graph of knowledge-based annotations (groups) of interest

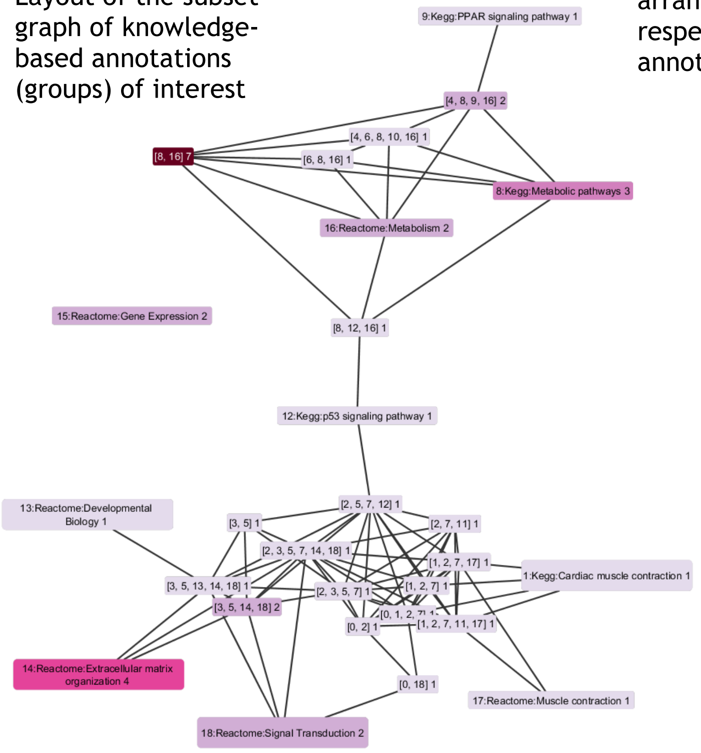

Lay out the subnetwork based on subset-graph to arrange nodes with respect to their annotations

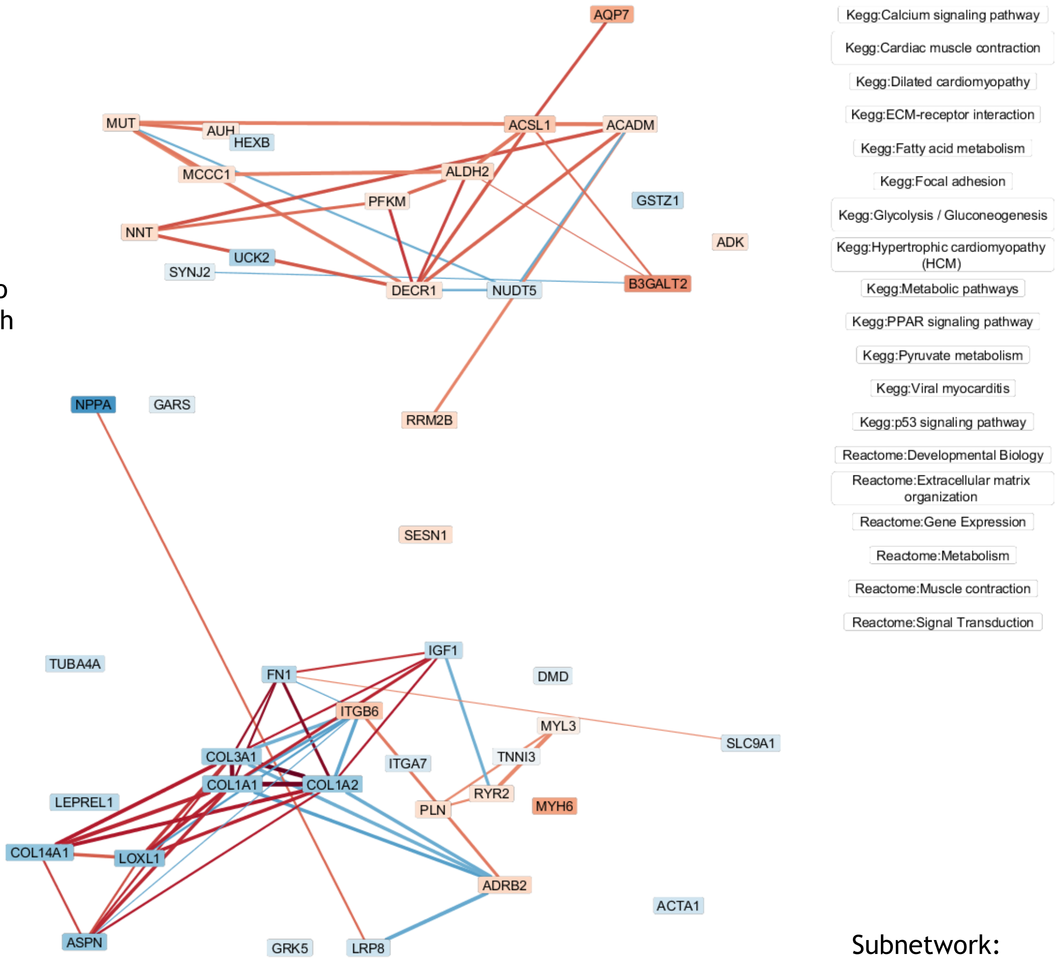

Subnetwork:
44 nodes
62 edges

Based on this layout and the evidence of change in a number of metabolic genes and extracellular matrix proteins, we could identify several genes that have not been previously characterized to play an important role in heart failure, although suspected to be relevant to LVEF improvement.

Selection of any annotation within the legend group (here extracellular matrix organization), highlights all nodes (genes) associated with this annotation.

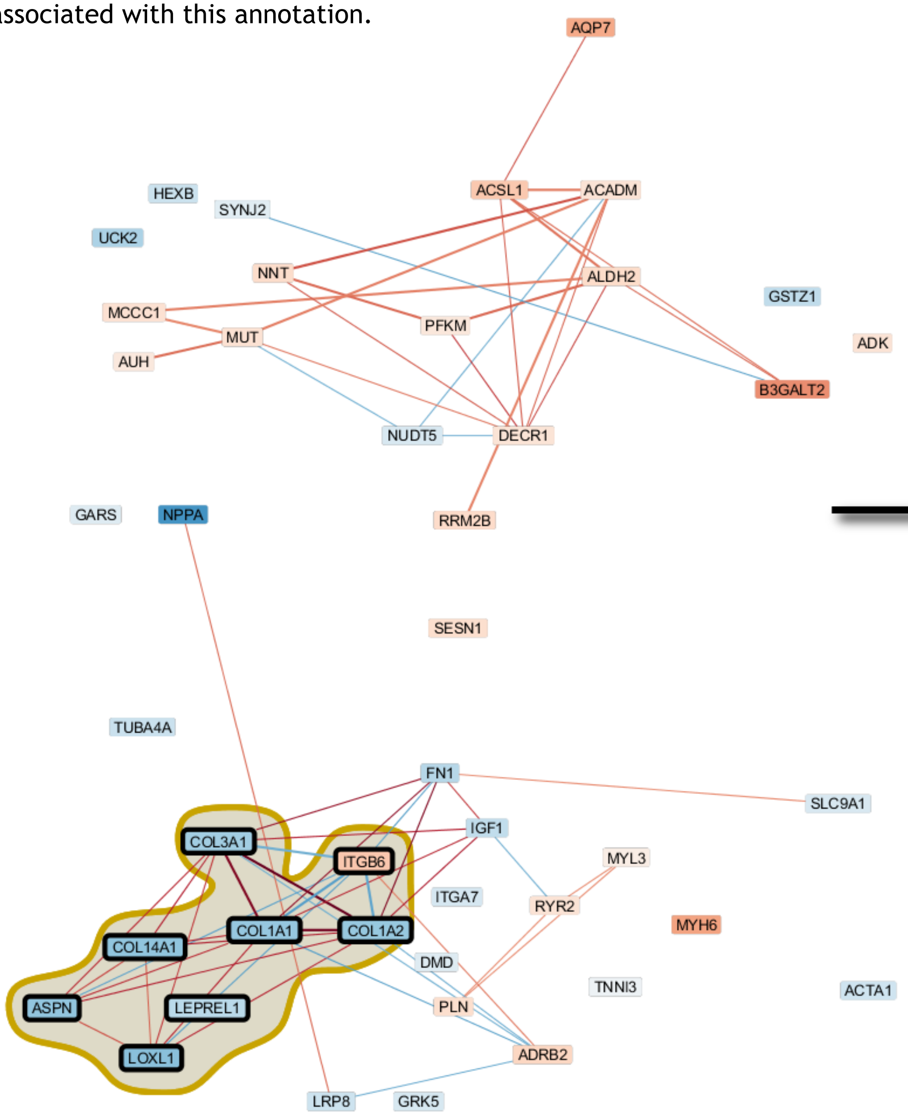

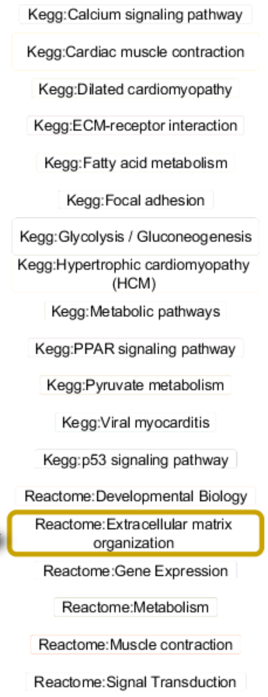

Open heat-map for genes associated with “extracellular matrix organization” to investigate differences in gene-expression between responders and non-responders

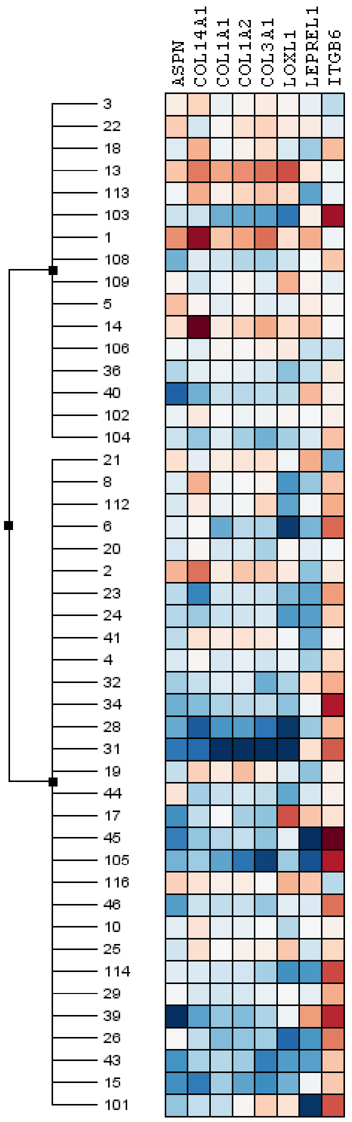

Selection of one or more annotation within the legend groups (here annotations related to metabolism), highlights all nodes (genes) associated with this annotation.

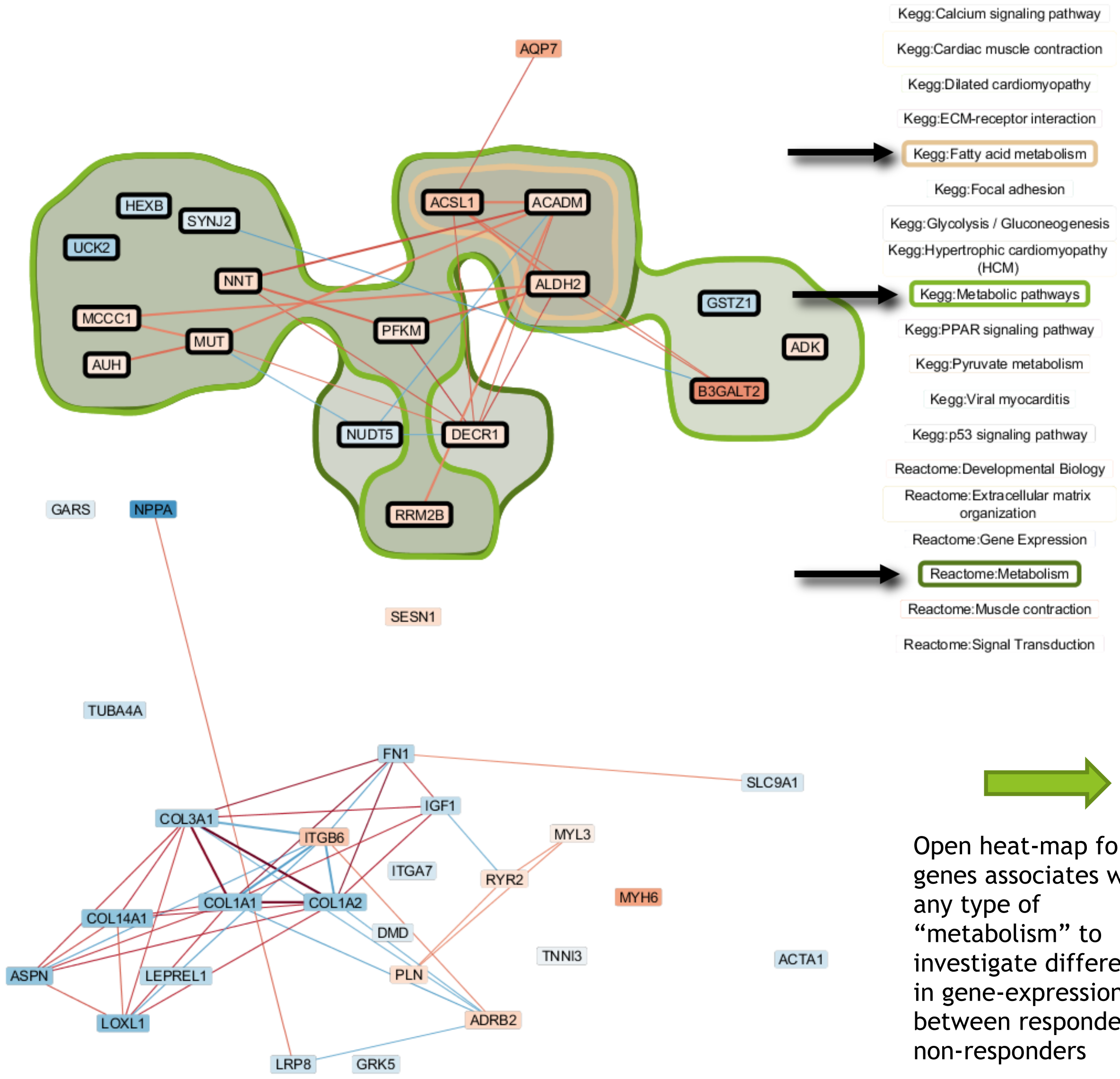

Open heat-map for genes associated with any type of “metabolism” to investigate differences in gene-expression between responders and non-responders

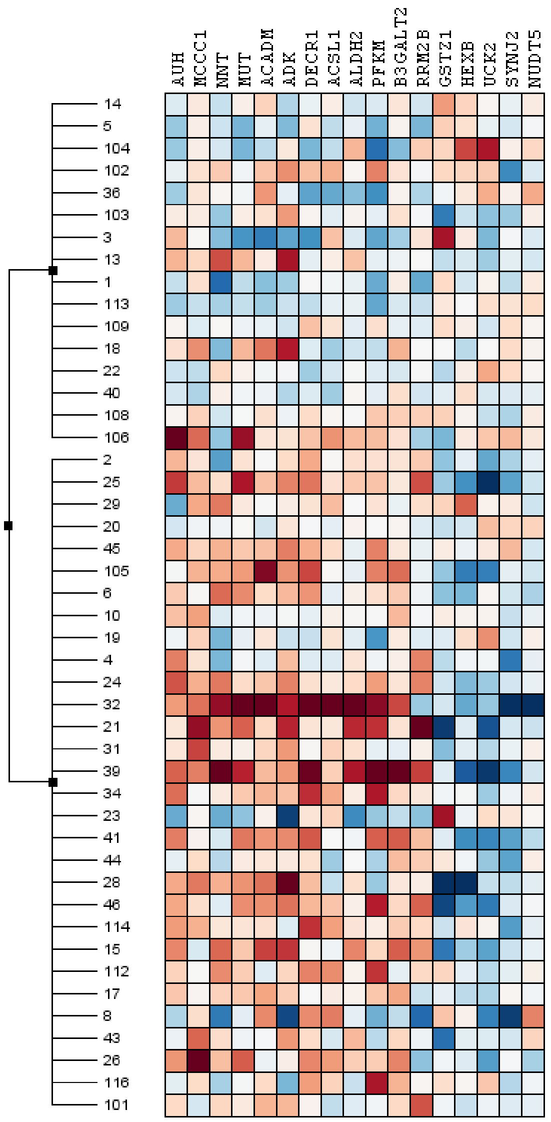

Selection of one or more annotation within the legend groups (here annotations related to muscle contraction), highlights all nodes (genes) associated with this annotation.

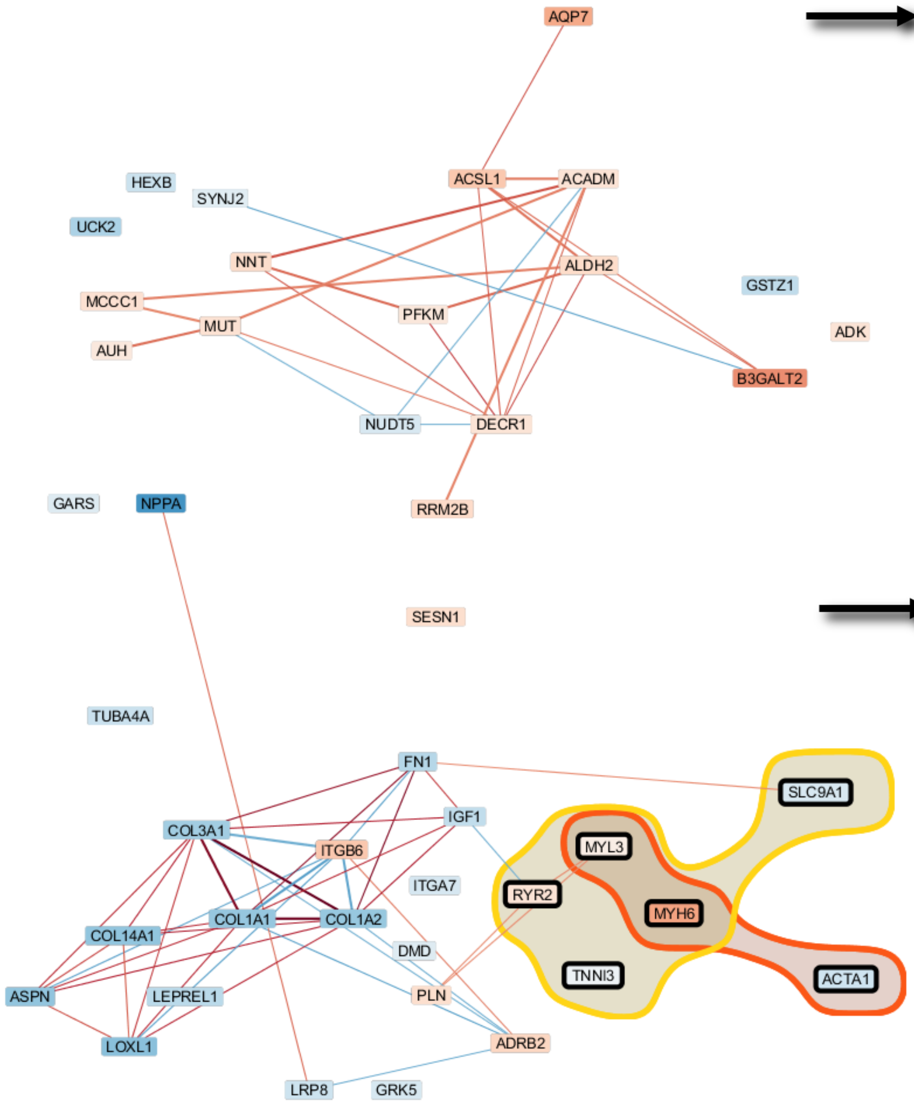

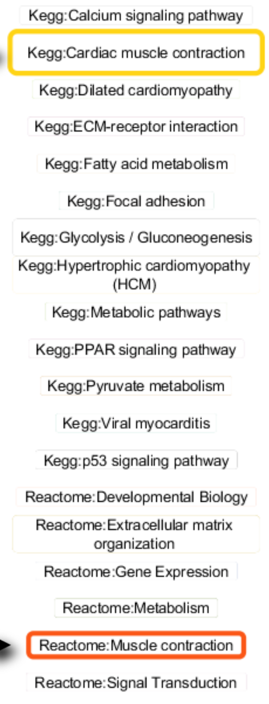

Open heat-map for genes associates with "muscle contraction" to investigate differences in gene-expression between responders and non-responders

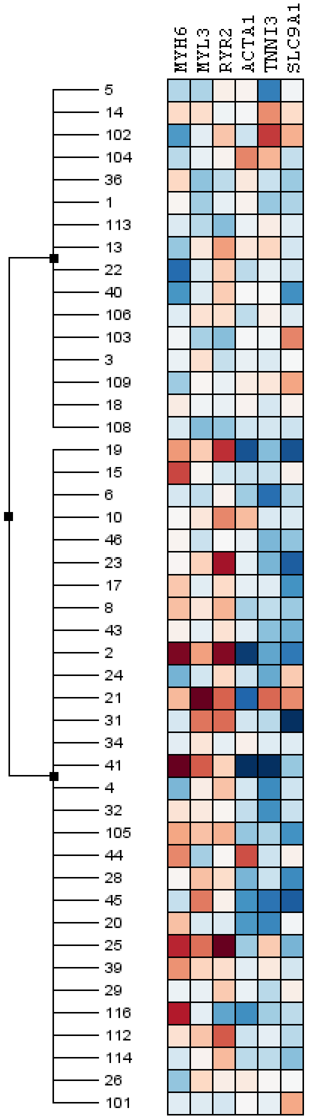

Supplement: Additional file 1 — Case Study 1. Detailed description of the analysis workflow of the first case study: Compare myocardial gene expression findings with components of the β1-adrenergic signaling network. [file 12859_2015_550_MOESM1_ESM.pdf]
