# Supplementary material for: Visual analysis of biological data-knowledge networks
Source: BMC Bioinformatics. 2015 Apr 29;16:135. doi: 10.1186/s12859-015-0550-z (PMC4456720; doi:10.1186/s12859-015-0550-z)

Case Study 2

Integrate and compare knowledge from expert sources to explore novel findings in experimental data

The data under investigation was derived from a study on “The Effect of ß-blockers on Structural Remodeling and Gene Expression in the Failing Human Heart” (BORG, NCT07989992).

Multiple statistical analyses identified expression of cholesterol modifying protein as being strongly associated with LVEF response. Not only was cholesterol trafficking not expected to have a role in recovery of heart function, but the gene product is the target of a new class of cholesterol-lowering medications currently in development, raising the possibility that these new drugs may impact heart failure. Therefore, we explored the plausibility and possible mechanisms of this novel hypothesis.

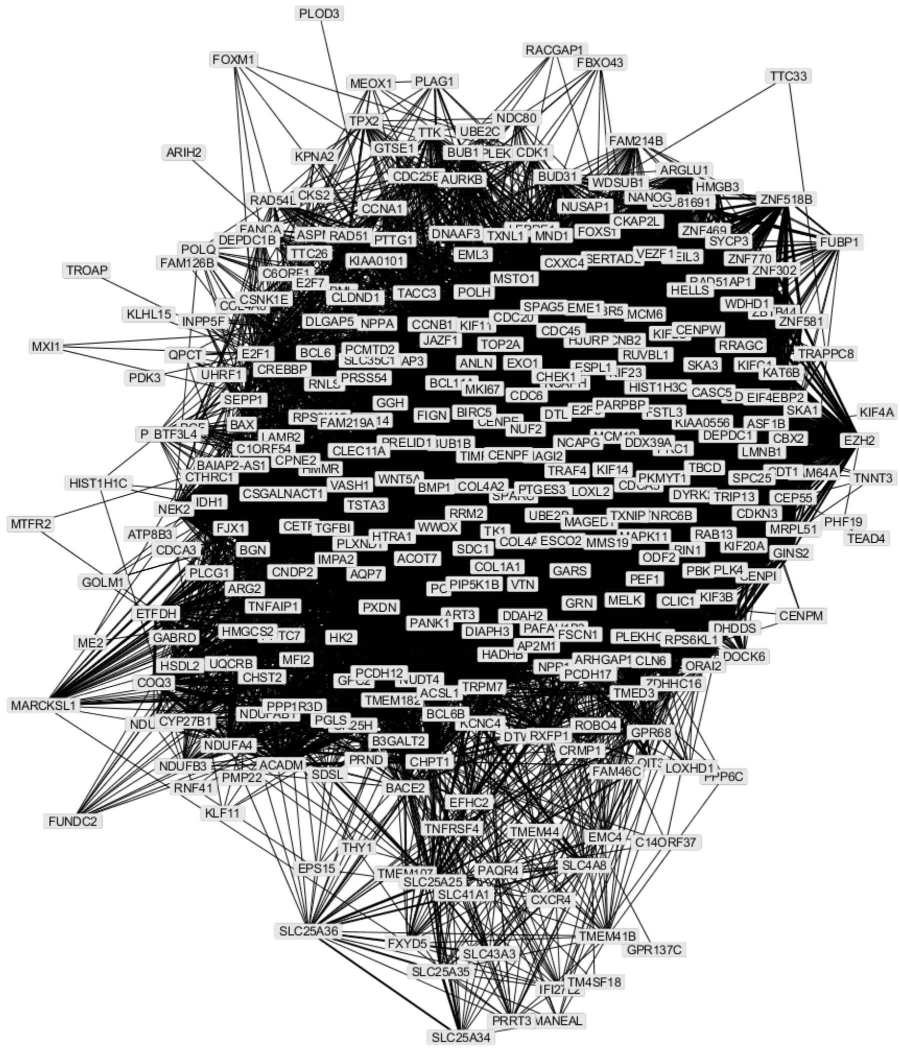

The combined data-knowledge network comprising 328 genes and 11081 relations between them.

Step1 - Filtering based on continuous DoI functions based on the experimental data: In order to focus on findings relevant to the primary analytical question, we first focused on the visualization of relevant experimental data. Therefore, we reduced the complexity of the data by filtering genes whose expression is associated with the phenotype – here LVEF response. In particular, we used the inverted statistical association of gene expression with LVEF response as DoI function – inverted as small p-values are of interest – that permitted dynamic filtering of nodes with low p-value (p-value<0.05). This way we could filter genes with a statistical difference in correlation between responders and non-responders. To focus on genes with evidence of meaningful correlation, we added a second DoI function allowing dynamic filtering of high gene-gene correlation values (|correlation|>0.5).

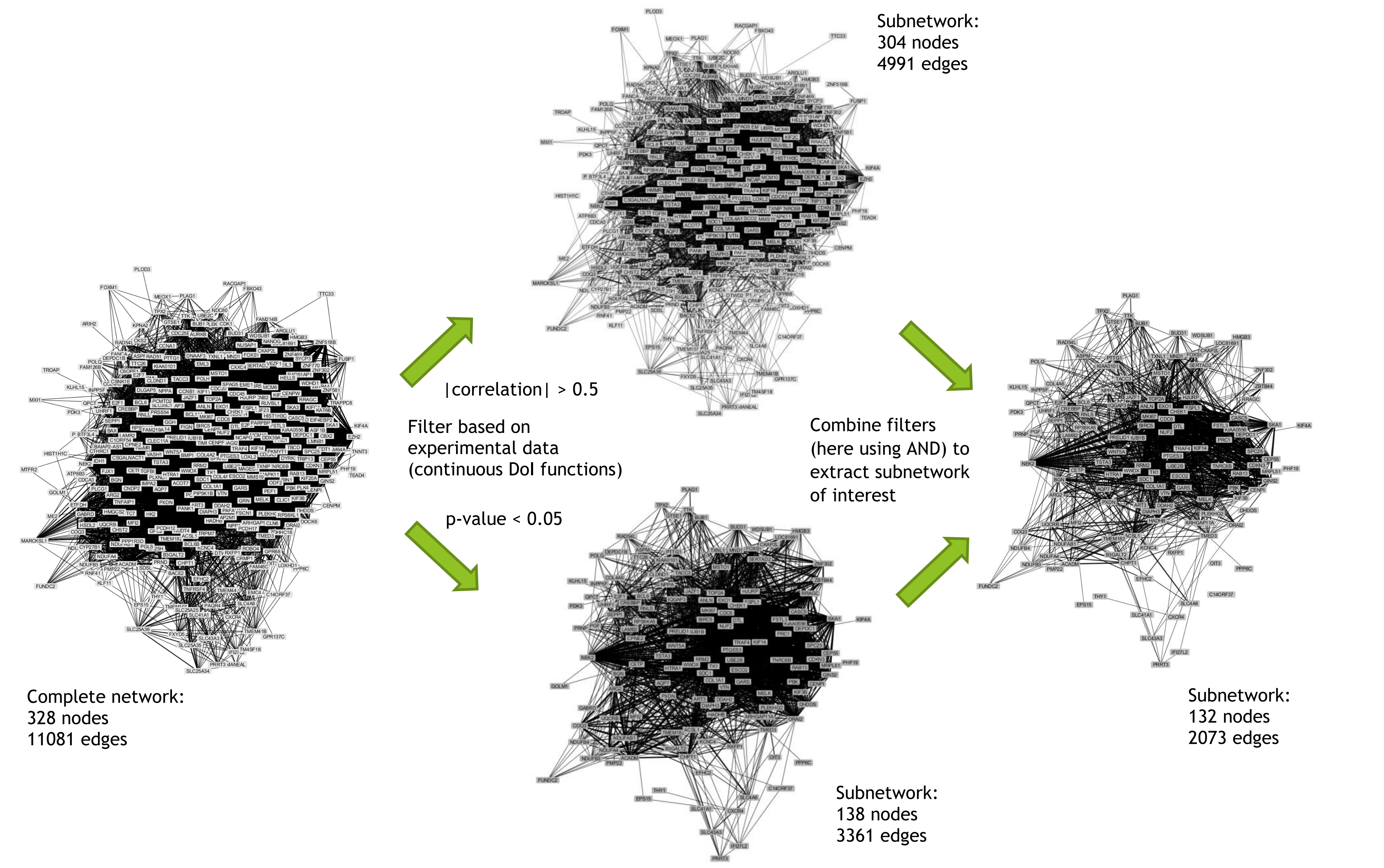

Step2 – Mapping of experimental data: The magnitude of change in gene expression and correlation was then mapped to the color of nodes and edges, respectively. Significantly up-regulated (down-regulated) genes show up as highly saturated red (blue) nodes. Gene expression correlation is mapped to color using the same color map, i.e., high positive (negative) correlations are mapped to a highly saturated red (blue).

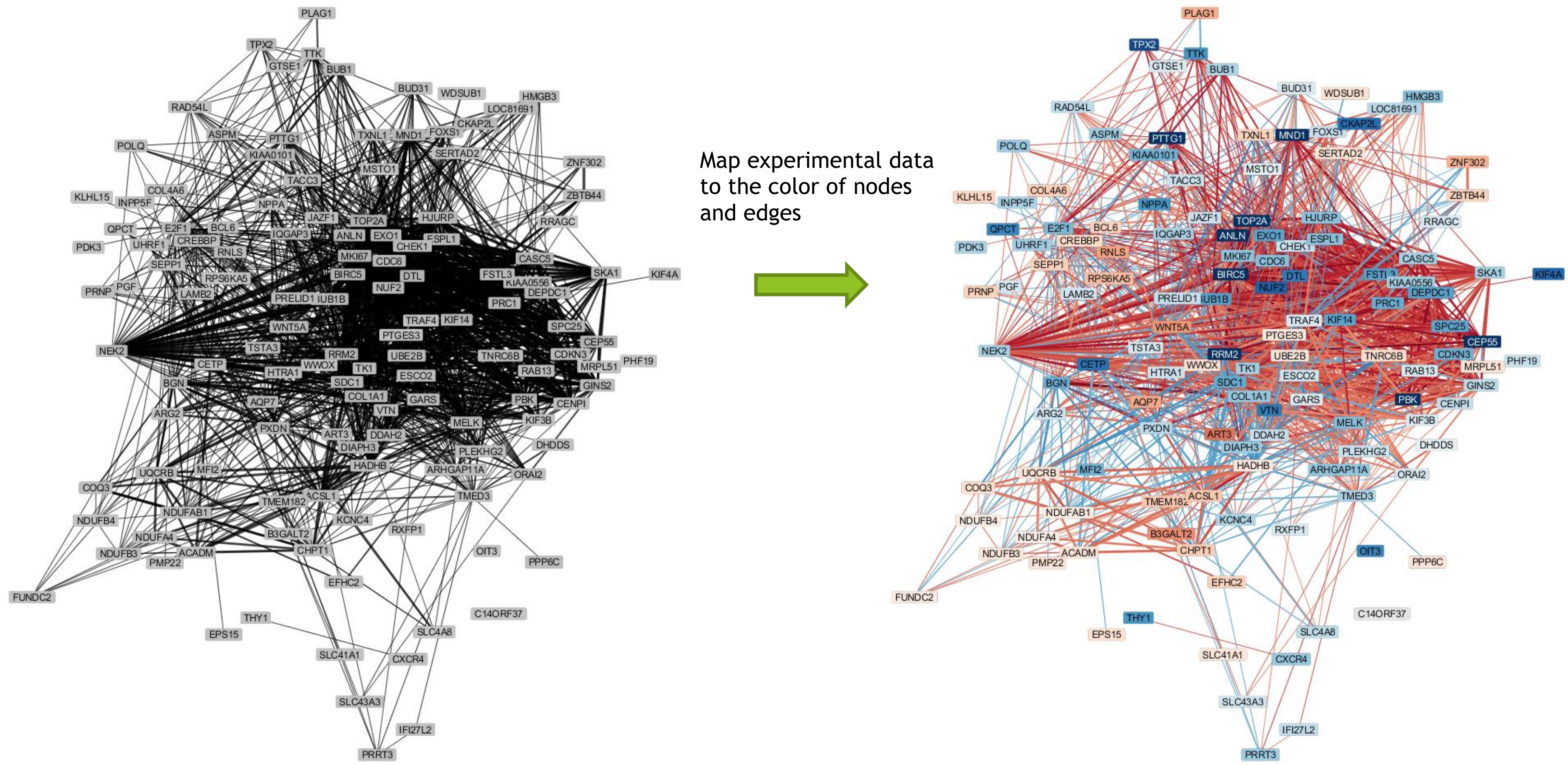

Step 3 - Filtering based on discrete DoI functions based on knowledge sources: In the first phase, we wanted to identify pathways associated with genes whose expression correlated with the cholesterol-modifying gene. Because essentially nothing is known about this cholesterol-modifying gene in heart failure, we identified genes with consistent pathway annotations. We created individual subnetworks based on similar terms from KEGG and Reactome. The KEGG pathways include: ECM-receptor interaction, fatty acid metabolism, focal adhesion, metabolic pathways, PPAR signaling pathway, regulation of actin cytoskeleton, and p53 signaling pathway.

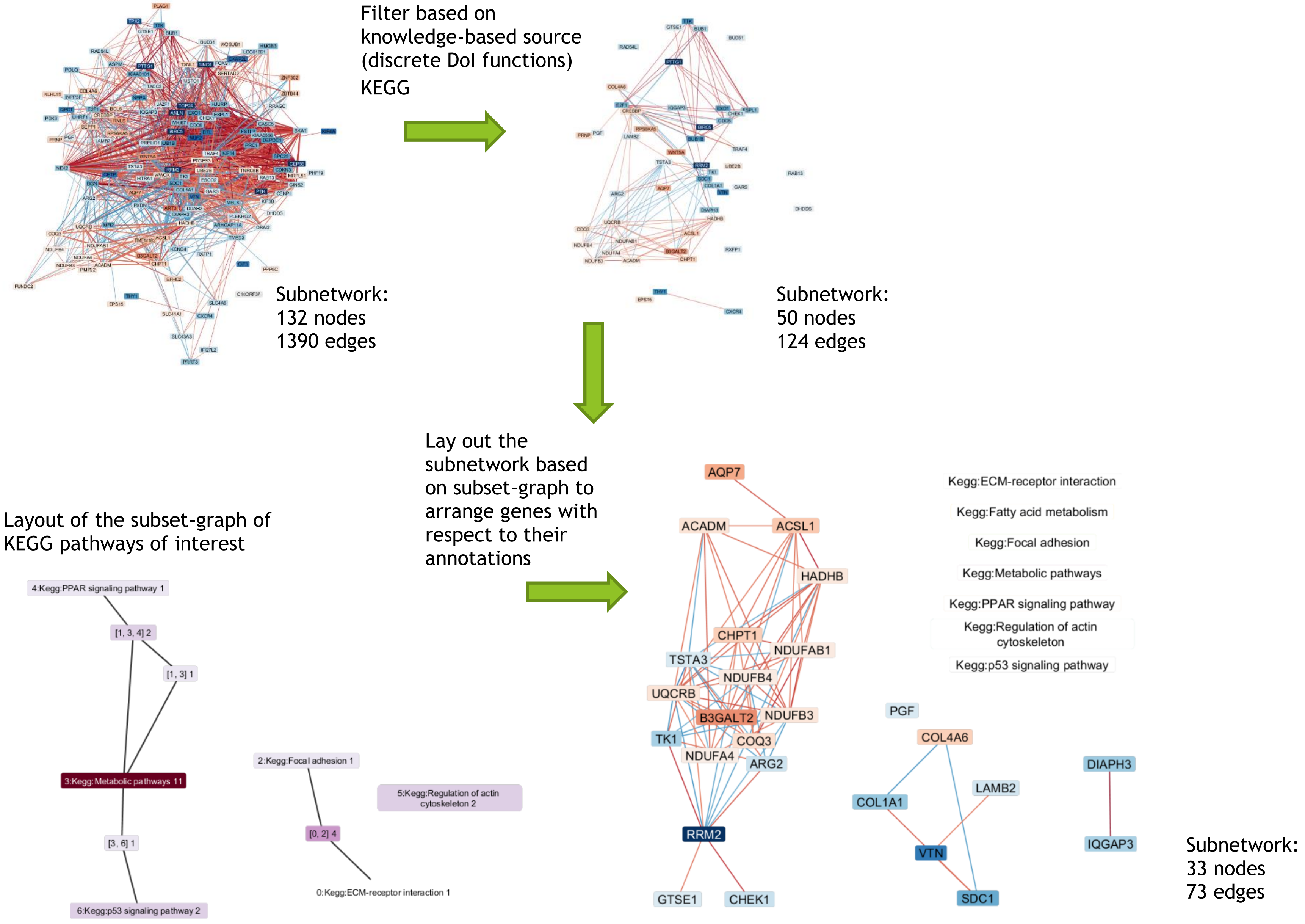

Highlight all Kegg annotations used for the layout as contours:

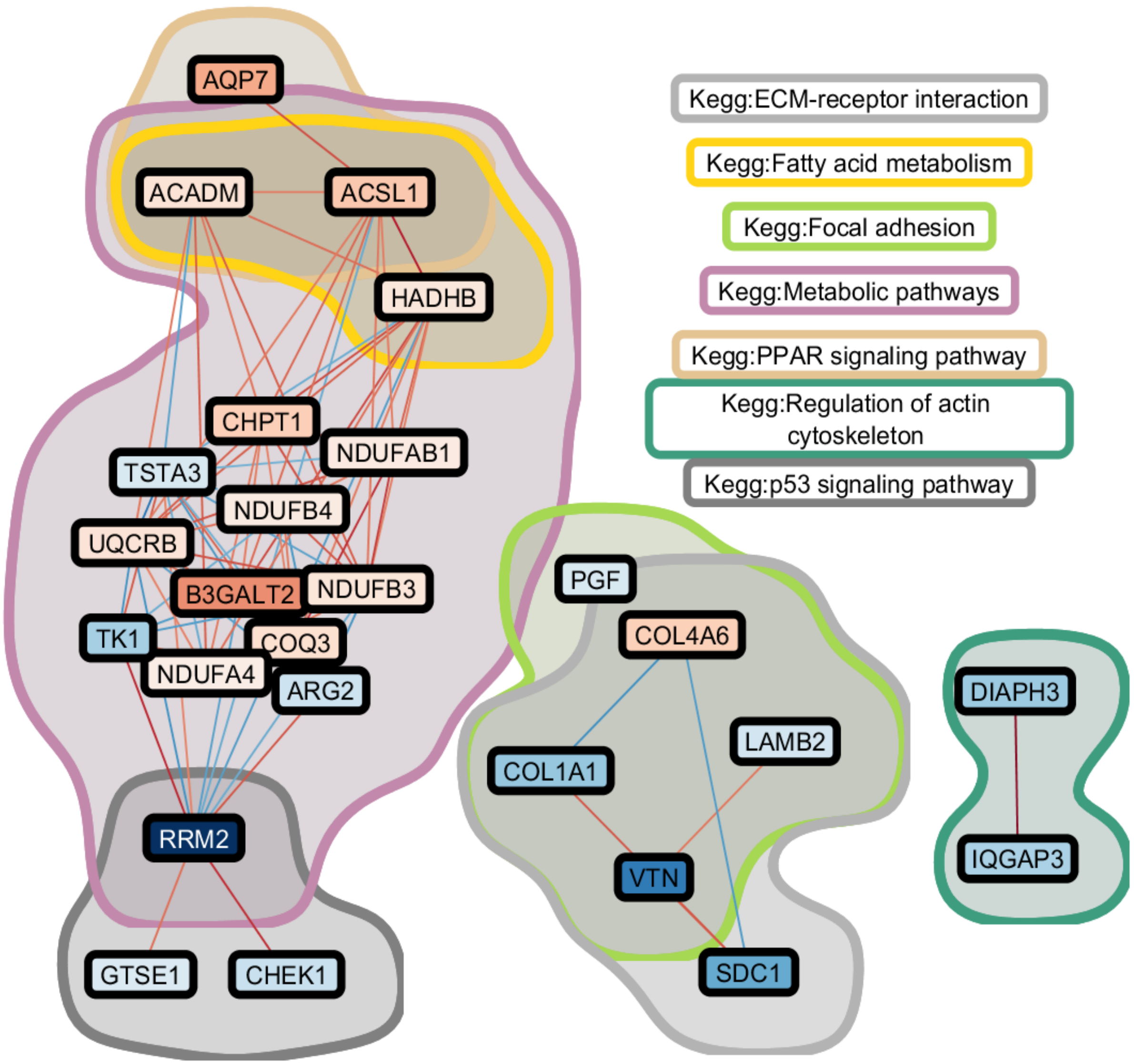

The Reactome pathways include: extracellular matrix organization, metabolism, and signal transduction.

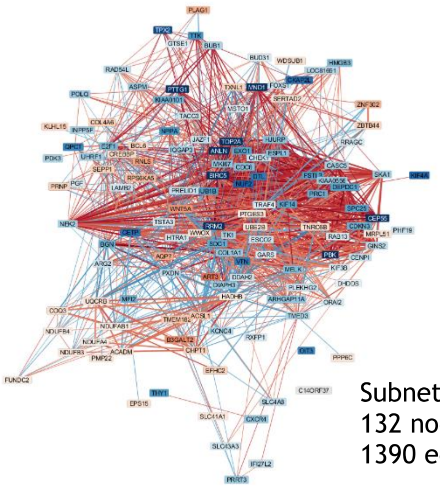

Subnetwork:
132 nodes
1390 edges

Filter based on
knowledge-based source
(discrete DoI functions)
Reactome

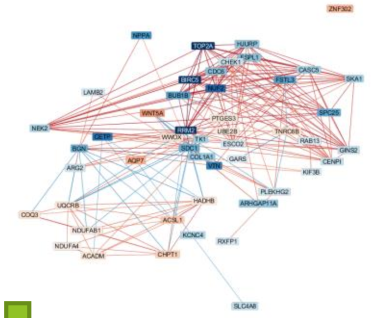

Subnetwork:
49 nodes
208 edges

Layout of the subset-graph of
Reactome pathways of interest

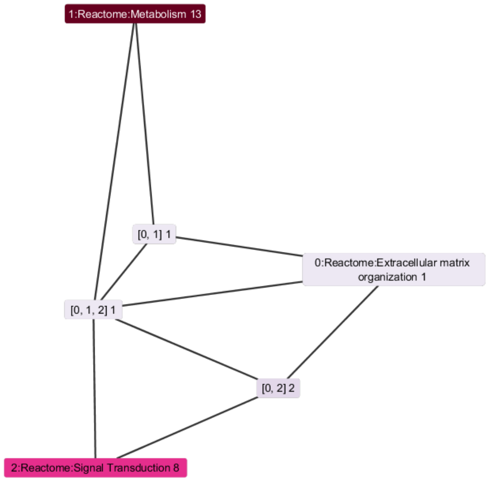

Lay out the
subnetwork based
on subset-graph to
arrange genes with
respect to their
annotations

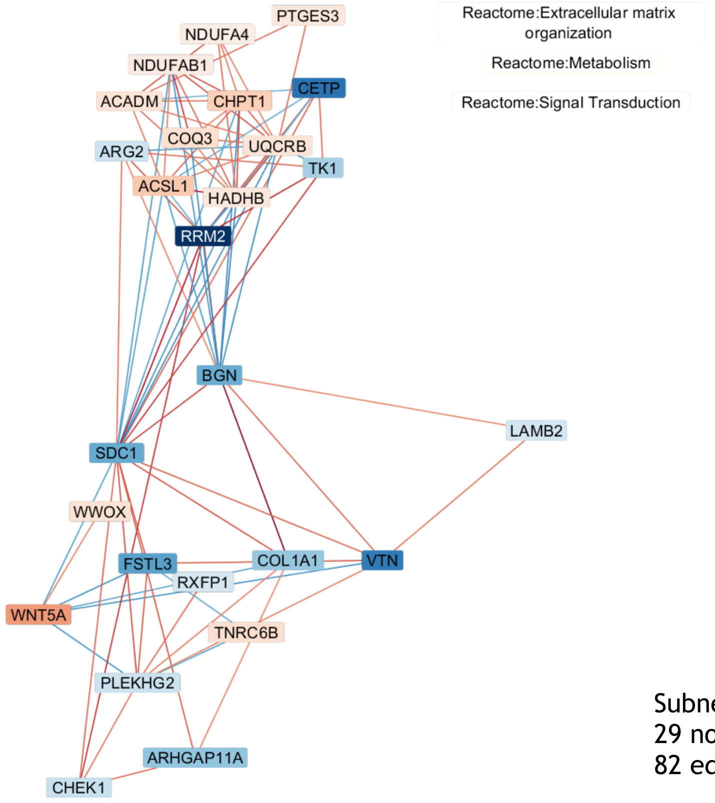

Subnetwork:
29 nodes
82 edges

Highlight all Reactome annotations used for the layout as contours:

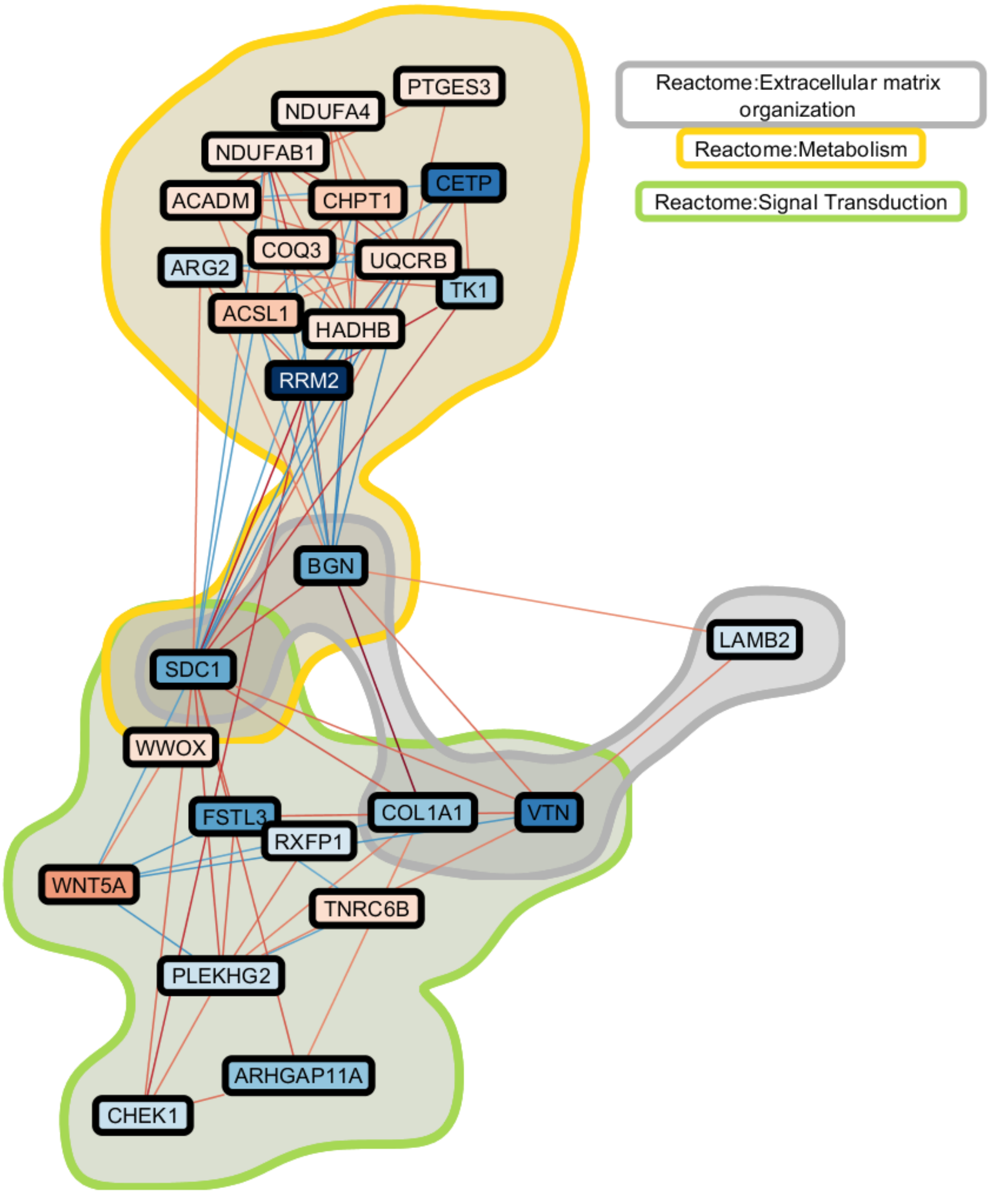

Step 4 - Compare combined data-knowledge networks derived using different knowledge sources: We created a combined and dynamically viewed gene annotations from each expert individually, in union, and the intersection between the two networks.

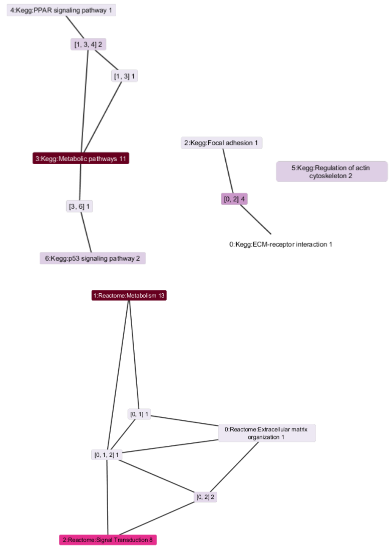

Layout of the subset-graphs of KEGG and Reactome.

Lay out the super-subset-graph to compare the two networks for KEGG and Reactome. The KEGG-based subnetwork is used as reference as it contains more nodes.

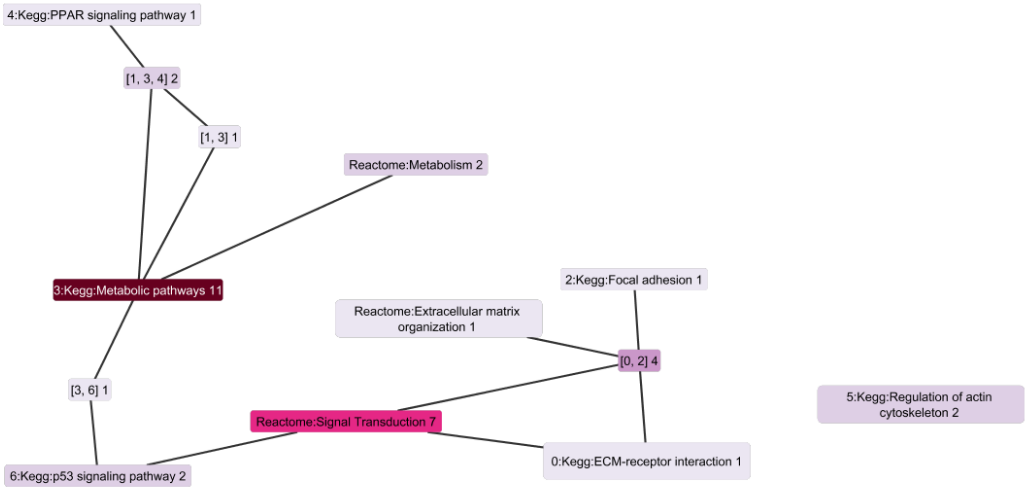

Lay out the super-graph to compare the two subnetworks for KEGG and Reactome.

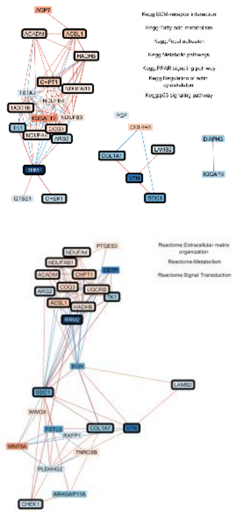

Layout of the subnetworks of KEGG and Reactome.

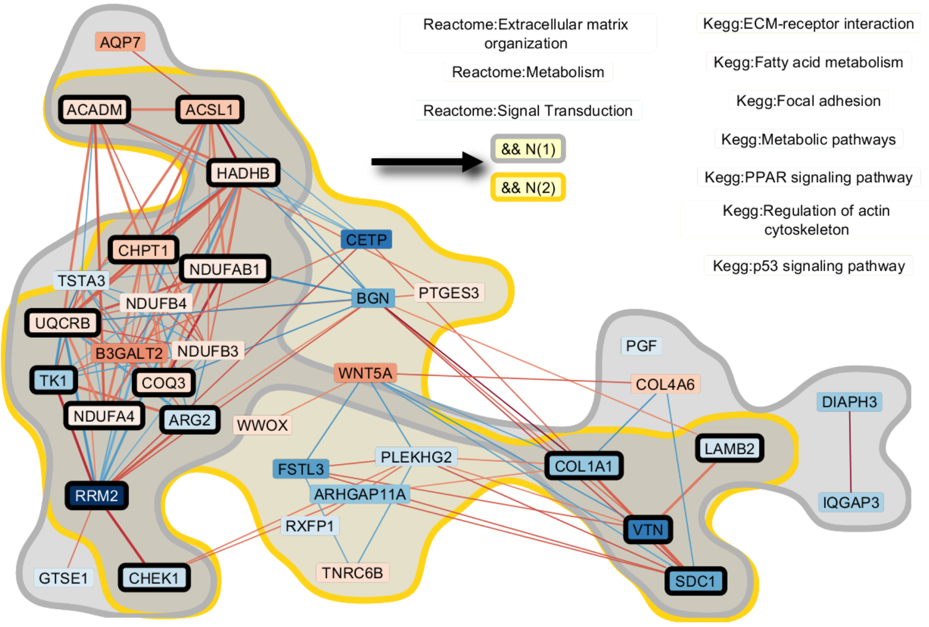

Super network of individual subnetworks: 48 nodes 113 edges

Labels for the two subnetworks are selected to highlight genes contained in both subnetworks; each set is surrounded by a contour.

Based on these operations we determined that expression of multiple extracellular matrix proteins and metabolic proteins according to both expert sources were correlated with LVEF response.

Selection of any annotation group (here extracellular matrix organization) within the legend, highlights all nodes (genes) associated with this annotation; the set of genes is surrounded by a contour.

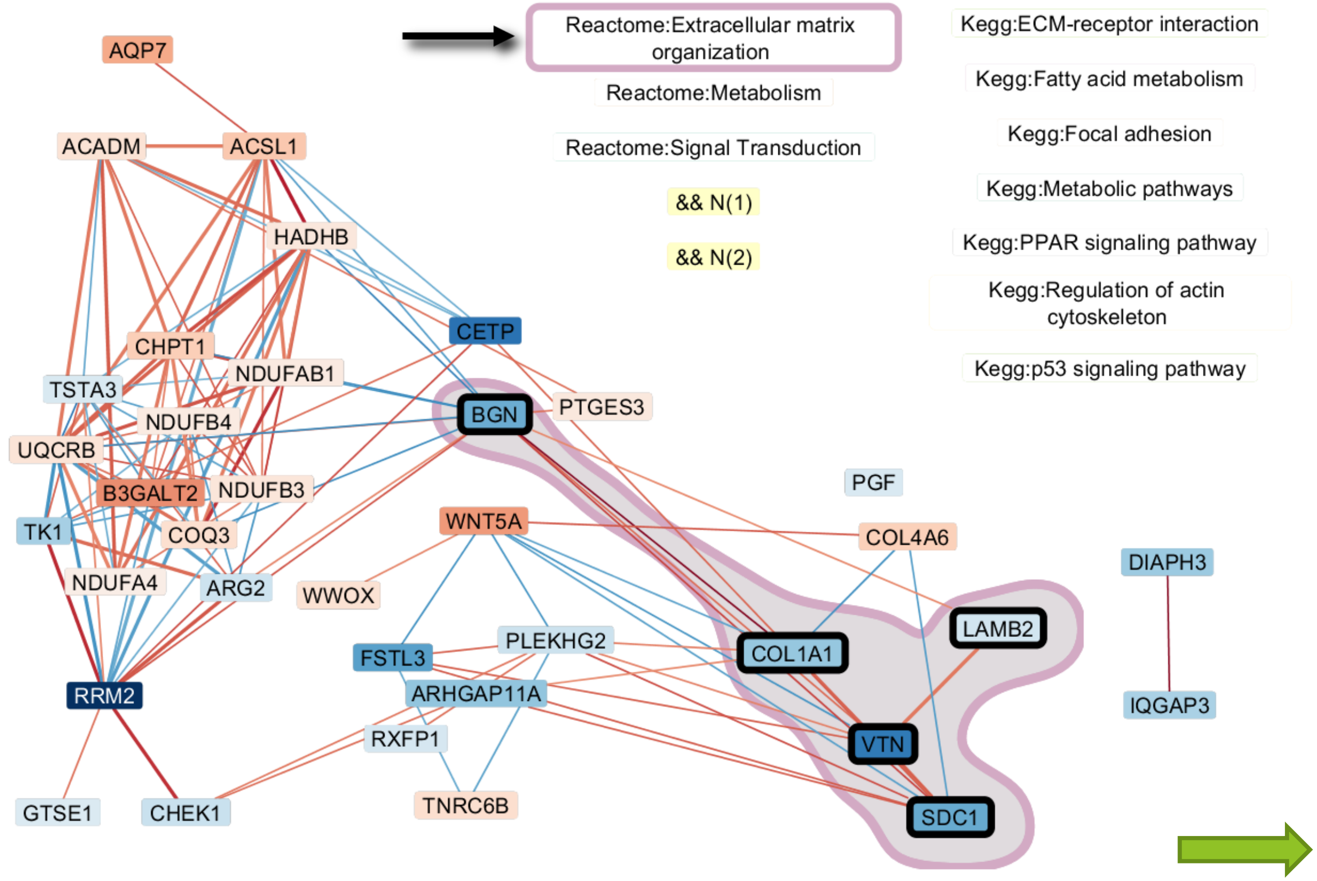

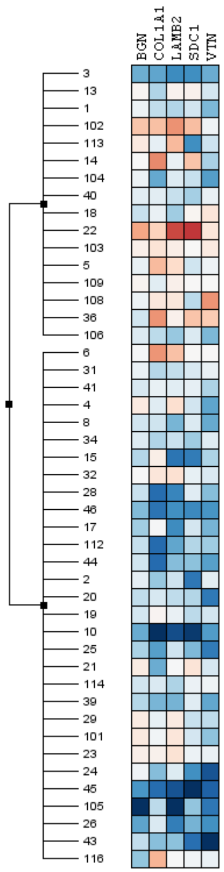

Open heat-map for genes associates with "extracellular matrix organization" to investigate differences in gene-expression between responders and non-responders

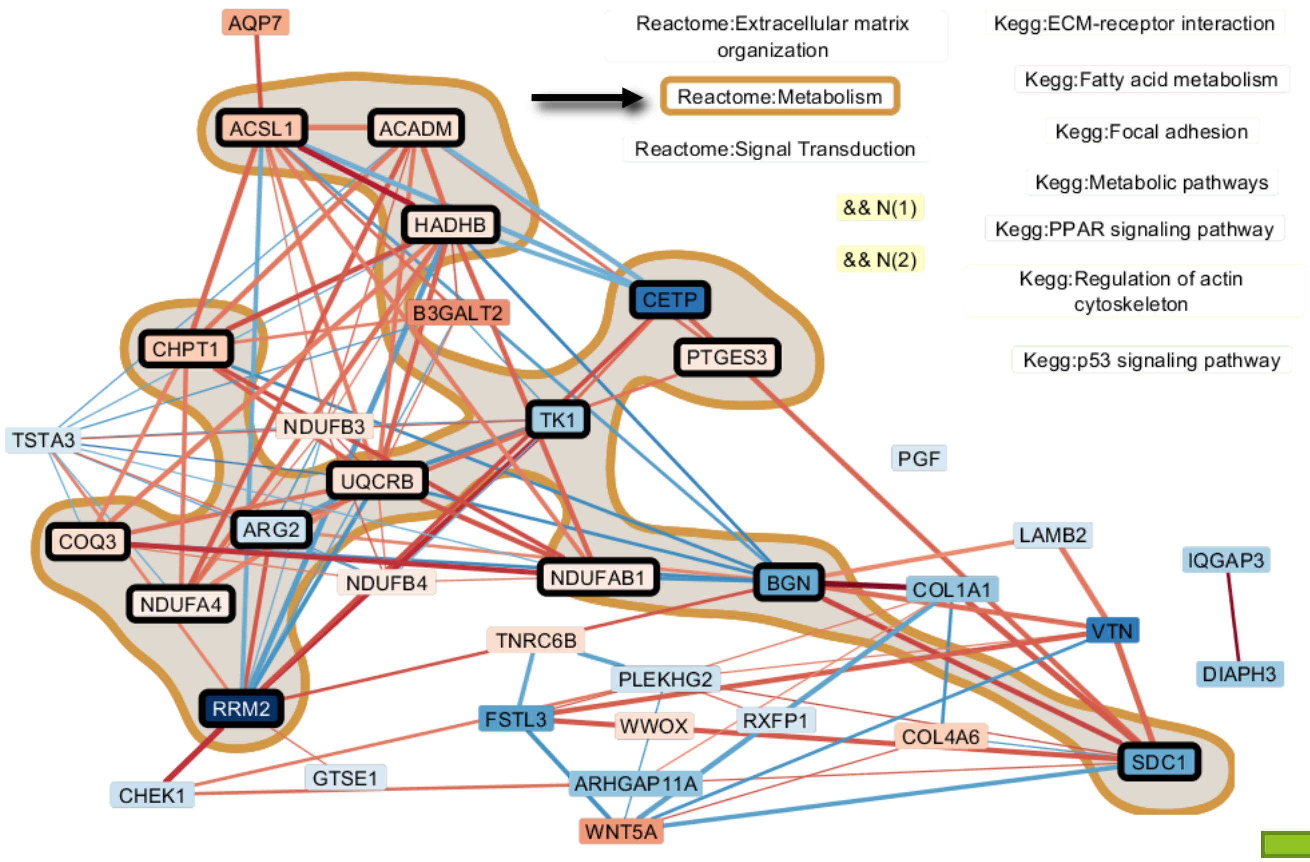

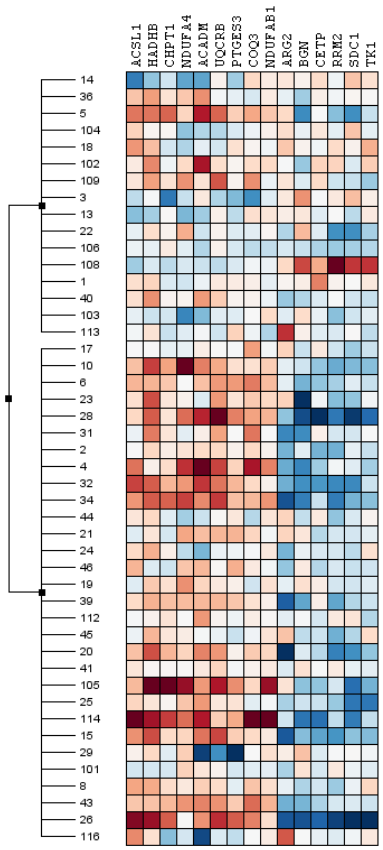

Open heat-map for genes associates with “metabolism” to investigate differences in gene-expression between responders and non-responders

Step 5 - Filtering based on discrete DoI functions based on knowledge sources: In the second phase, explored relationships between correlated genes along multiple axes we combine distinct networks (Task IV). We visualize both the cellular localization of each gene as well as their functional/pathway annotations by creating subnetworks using the Gene-Ontology Cellular Compartment expert in combination with Reactome functional annotations. For the Gene-Ontology Cellular Compartment expert, we chose the following terms of interest: basal lamina, basement membrane, collagen type I, endoplasmic reticulum membrane, extracellular space, proteinaceous extracellular matrix, and sarcolemma.

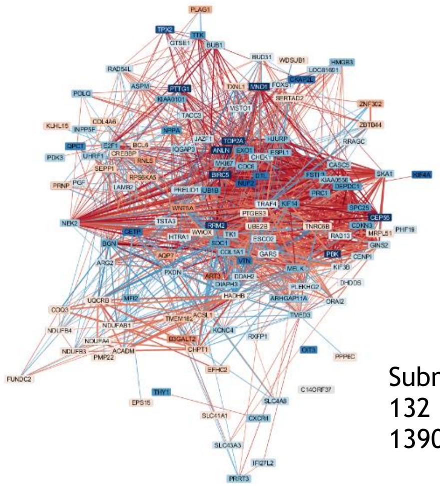

Subnetwork:
132 nodes
1390 edges

Filter based on knowledge-based source (discrete DoI functions)
Gene-Ontology CC

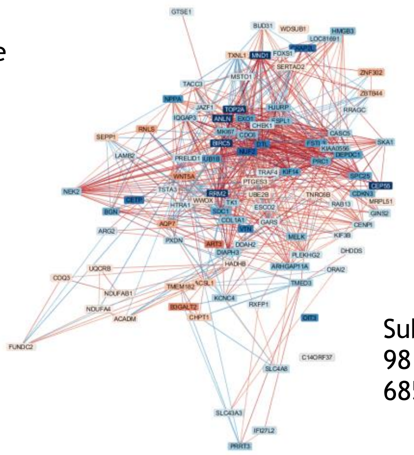

Subnetwork:
98 nodes
685 edges

Lay out the subnetwork based on subset-graph to arrange genes with respect to their annotations

Layout of the subset-graph of GO-CC terms of interest

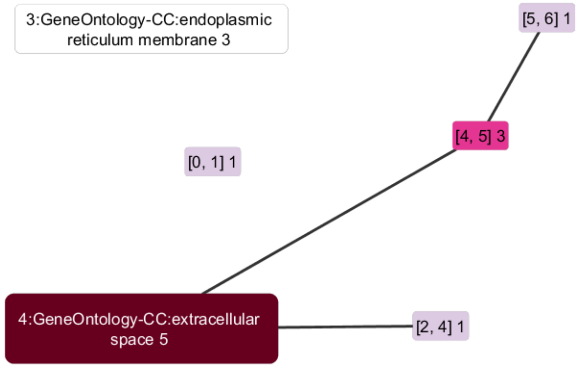

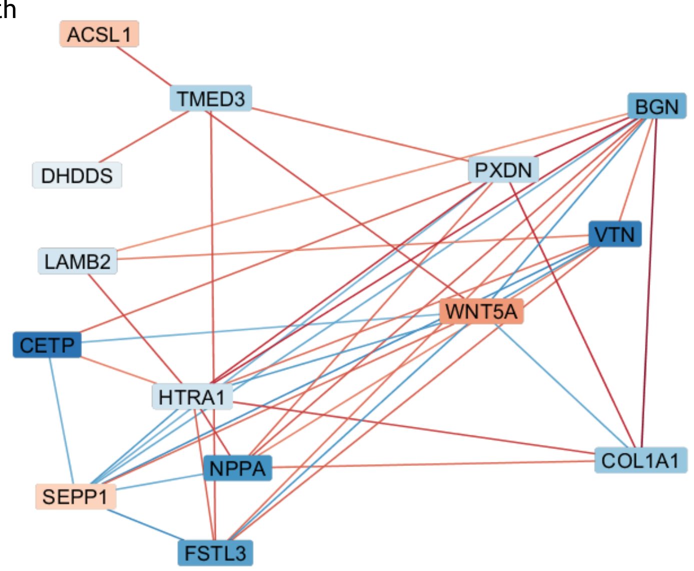

GeneOntology-CC:basal lamina
GeneOntology-CC:basement membrane
GeneOntology-CC:collagen type I
GeneOntology-CC:endoplasmic reticulum membrane
GeneOntology-CC:extracellular space
GeneOntology-CC:proteinaceous extracellular matrix
GeneOntology-CC:sarcolemma

Subnetwork:
21 nodes
39 edges

Highlight all GeneOntology-CC annotations used for the layout as contours:

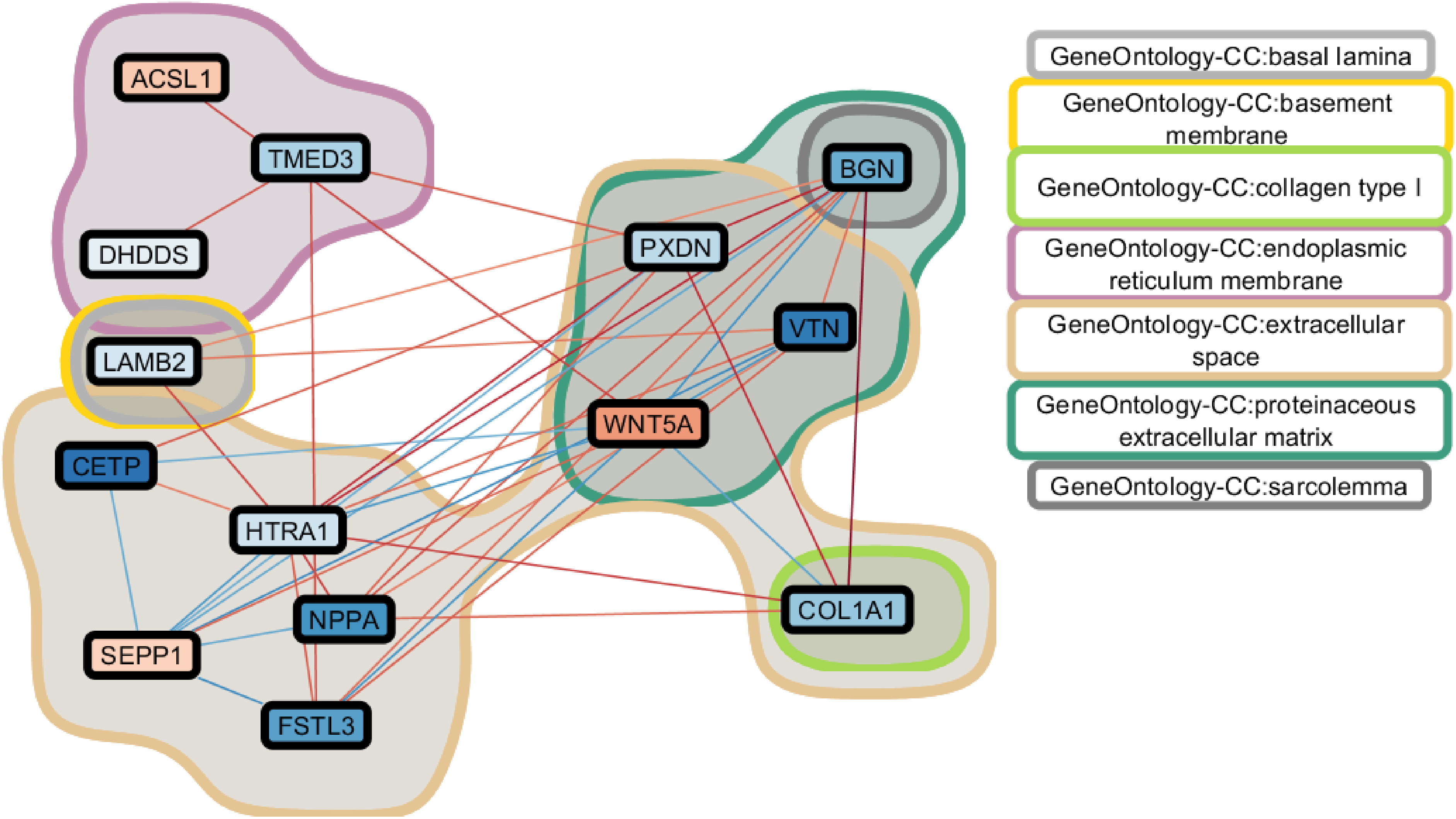

The Reactome pathways include: extracellular matrix organization, gene expression, metabolism, and signal transduction.

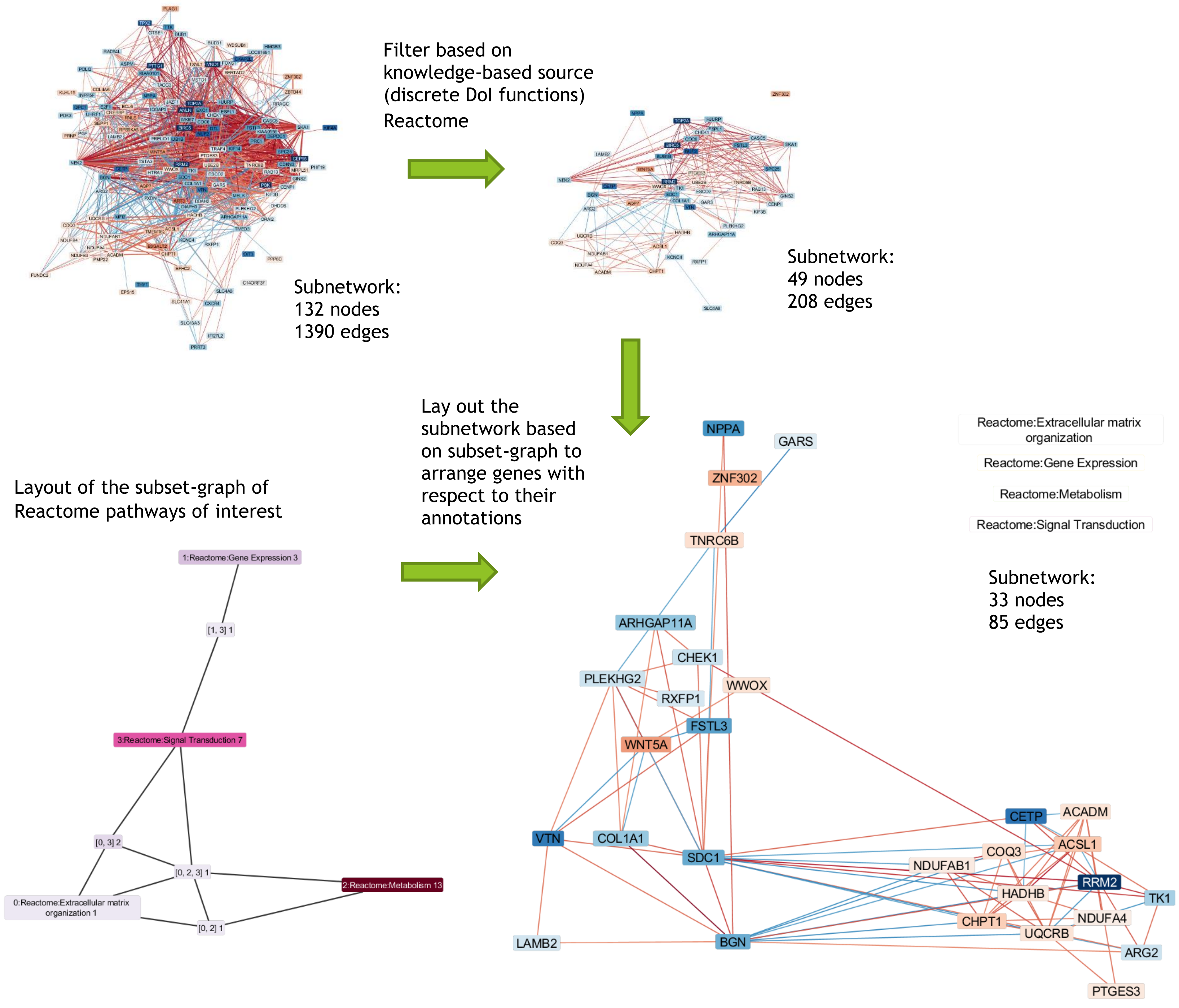

Highlight all GeneOntology-CC annotations used for the layout as contours:

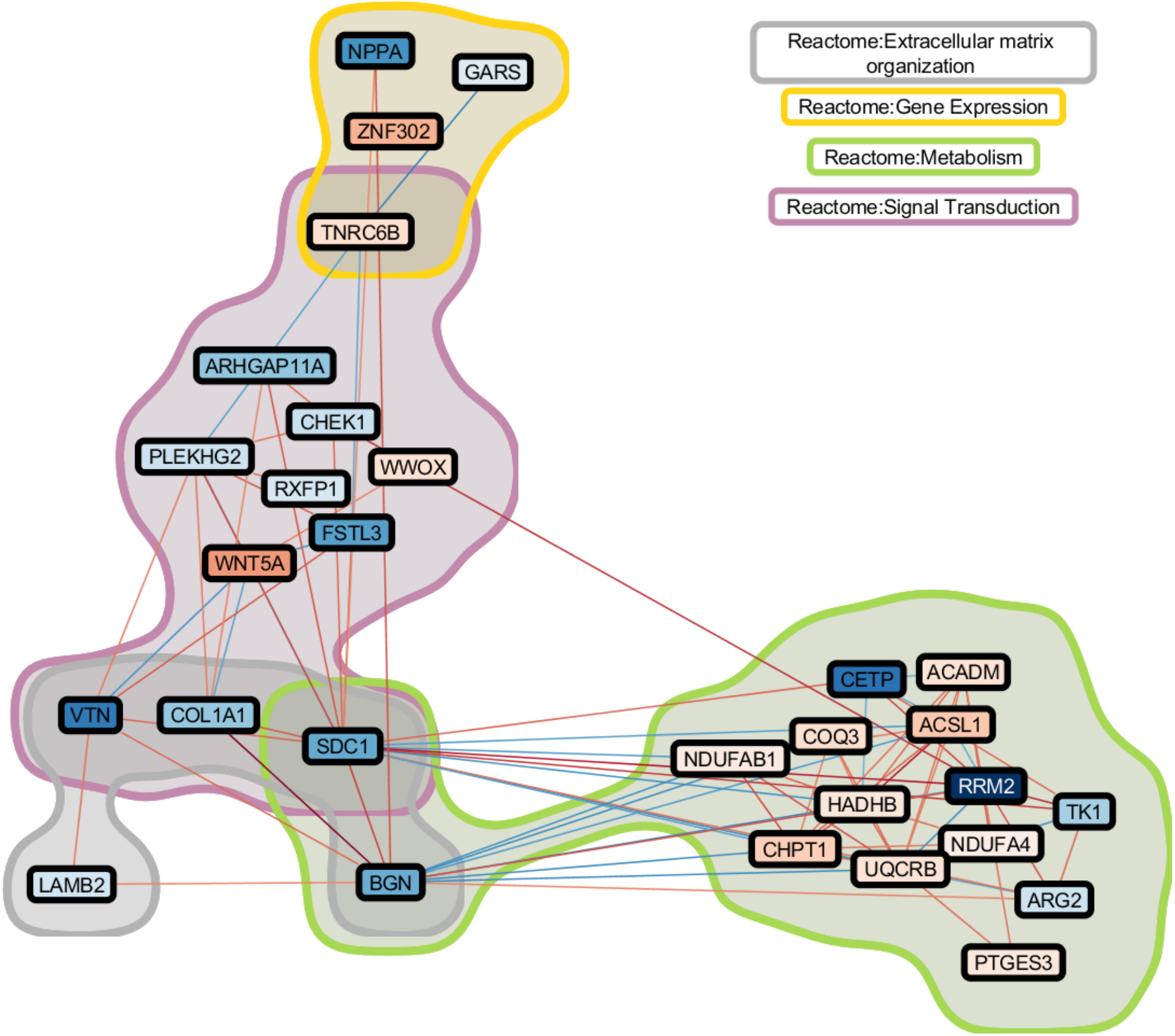

Step 6 - Compare combined data-knowledge networks derived using different knowledge sources: By exploring the overlap between these two experts in a combined network, we found clusters of correlated genes within specific compartments, within specific pathways, and functionally related genes localized to the same compartment.

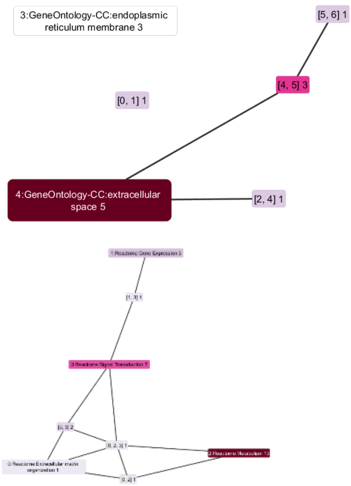

Layout of the subset-graphs for Reactome and Gene-Ontology CC.

Lay out the super-subset-graph to compare the two networks for Reactome and Gene-Ontology CC. The Reactome-based subnetwork is used as reference as it contains more nodes.

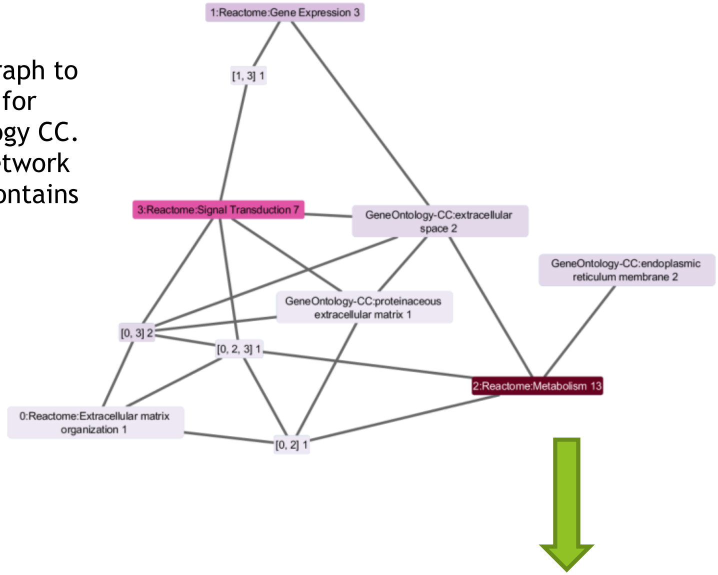

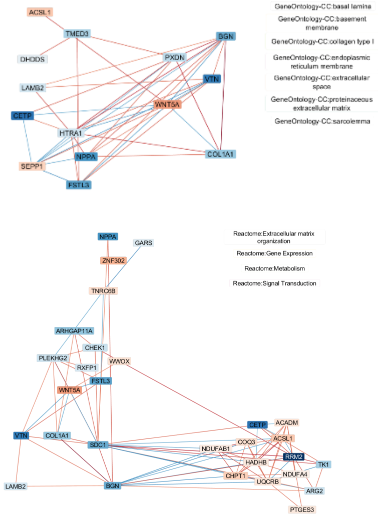

Layout of the subnetworks for Reactome and Gene-Ontology CC.

Lay out the super-graph to compare the two subnetworks for Reactome and Gene-Ontology CC.

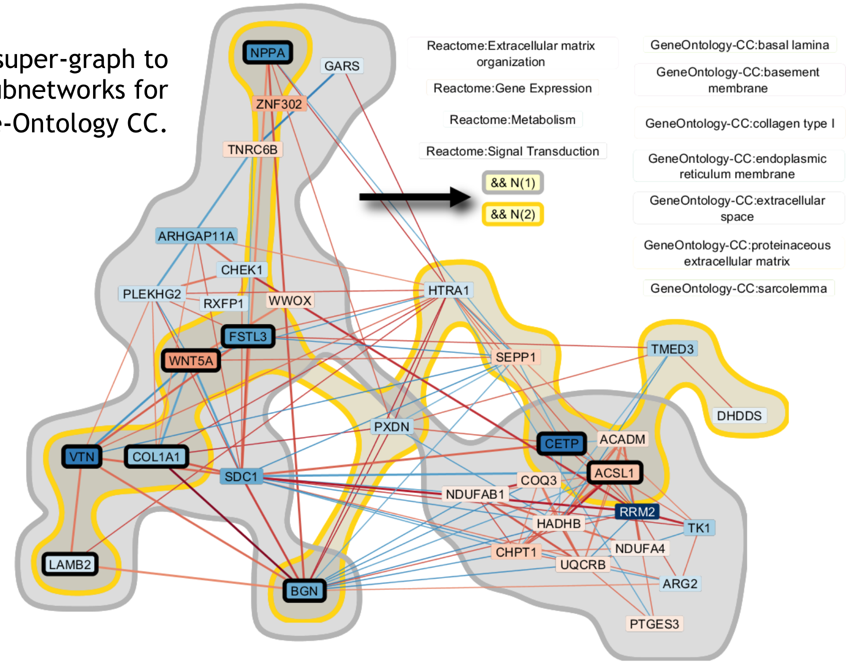

Labels for the two subnetworks are selected to highlight genes contained in both subnetworks. Each set of nodes (for each subnetwork) is surround by a filled contour.

Super network of individual subnetworks: 47 nodes 119 edges

These findings can (a) support confirmatory translational experiments by identifying specific candidate genes in specific cellular compartments to isolate and (b) identify genes with extracellular products (such as NPPA or CETP) that might be used as diagnostics in peripheral circulation. Incorporation of pharmaceutical target experts would also allow the identification of candidate therapeutic targets to support drug repurposing or novel drug application development.

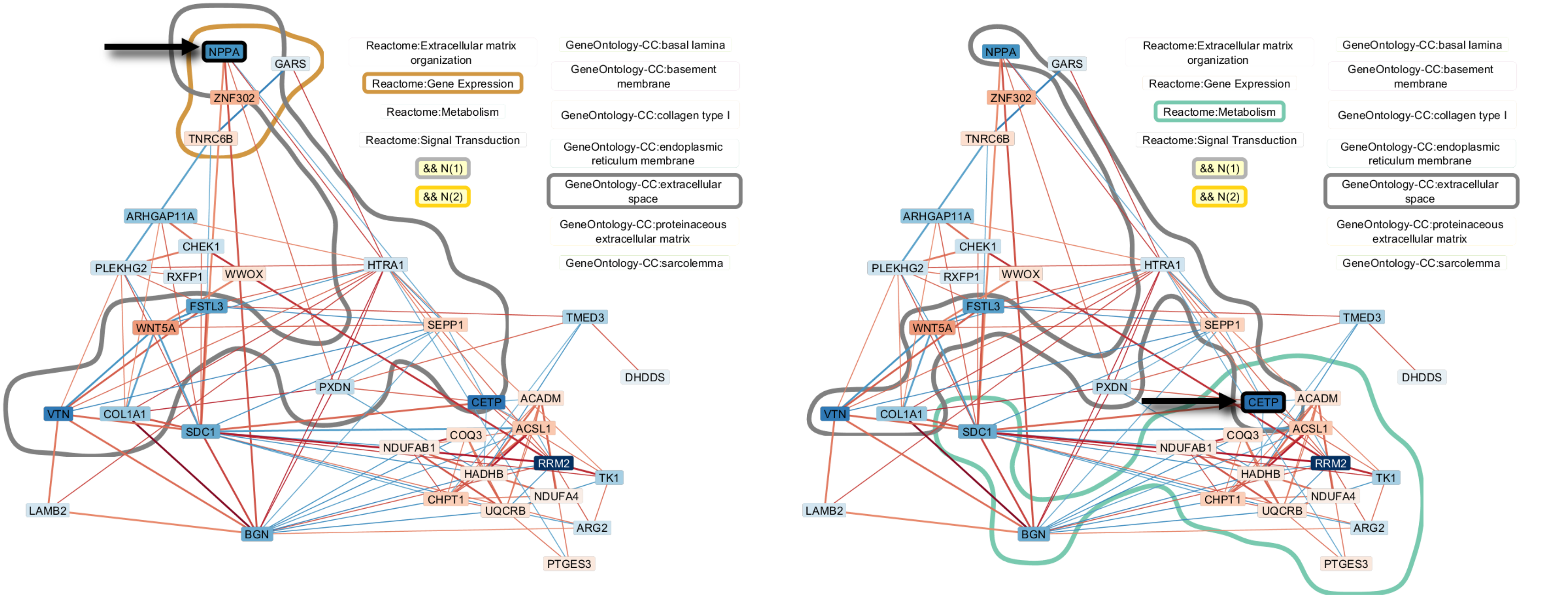

The selection of a gene (here NPPA and CETP) highlights the associated annotations within the legend and shows the contours of the sets (in this case not filled) of the associated annotations.

The selection of the GO-CC term 'extracellular space' highlights all nodes of that network that are associated with this term (e.g., NPPA and CETP) and surrounds them by a filled contour.

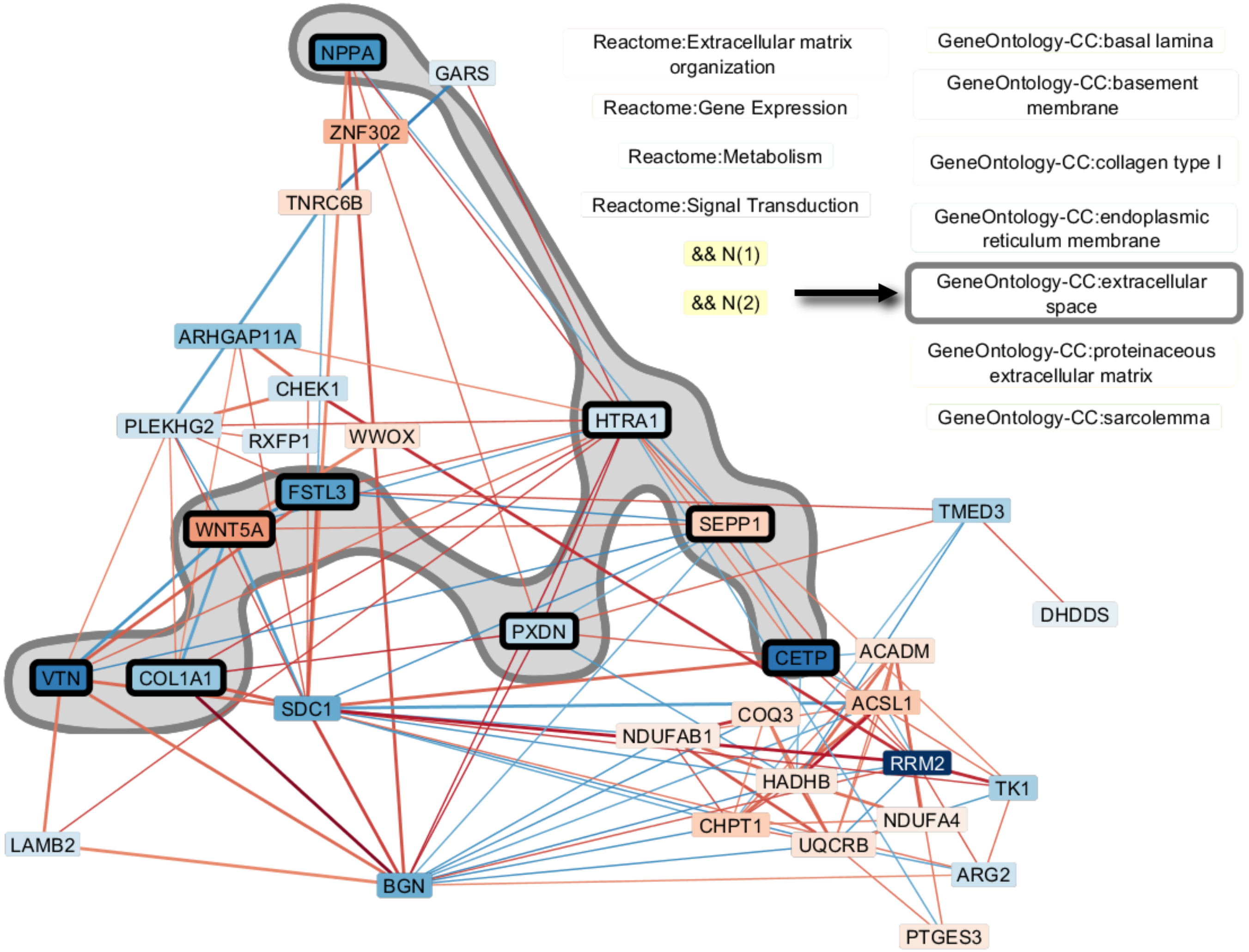

Supplement: Additional file 2 — Case Study 2. Detailed description of the analysis workflow of the second case study: Integrate and compare knowledge from expert sources to explore novel findings in experimental data. [file 12859_2015_550_MOESM2_ESM.pdf]
